# Scaling down the dimensions of a Fabry–Perot polymer film acoustic sensor for photoacoustic endoscopy

**DOI:** 10.1117/1.JBO.29.S1.S11514

**Published:** 2024-01-02

**Authors:** Tong Li, Tse-Shao Chang, Ahmad Shirazi, Xiaoli Wu, Wei-Kuan Lin, Ruoliu Zhang, Jay L. Guo, Kenn R. Oldham, Thomas D. Wang

**Affiliations:** a University of Michigan, Department of Mechanical Engineering, Ann Arbor, Michigan, United States; b University of Michigan, Division of Integrative Systems and Design, Ann Arbor, Michigan, United States; c University of Michigan, Department of Internal Medicine, Division of Gastroenterology, Ann Arbor, Michigan, United States; d University of Michigan, Department of Electrical and Computer Engineering, Ann Arbor, Michigan, United States; e University of Michigan, Department of Biomedical Engineering, Ann Arbor, Michigan, United States; f University of Michigan, Department of Macromolecular Science and Engineering, Ann Arbor, Michigan, United States; g University of Michigan, Department of Applied Physics, Ann Arbor, Michigan, United States

**Keywords:** sensor, interferometer, imaging, photoacoustic, transfer matrix, finite-element analysis

## Abstract

**Significance:**

A Fabry–Perot (FP) polymer film sensor can be used to detect acoustic waves in a photoacoustic endoscope (PAE) if the dimensions can be adequately scaled down in size. Current FP sensors have limitations in size, sensitivity, and array configurability.

**Aim:**

We aim to characterize and demonstrate the imaging performance of a miniature FP sensor to evaluate the effects of reduced size and finite dimensions.

**Approach:**

A transfer matrix model was developed to characterize the frequency response of a multilayer miniature FP sensor. An analytical model was derived to describe the effects of a substrate with finite thickness. Finite-element analysis was performed to characterize the temporal response of a sensor with finite dimensions. Miniature $2\times 2\;{\mathrm{mm}}^{2}$ FP sensors were designed and fabricated using gold films as reflective mirrors on either side of a parylene C film deposited on a glass wafer. A single-wavelength laser was used to interrogate the sensor using illumination delivered by fiber subprobes. Imaging phantoms were used to verify FP sensor performance, and *in vivo* images of blood vessels were collected from a live mouse.

**Results:**

The finite thickness substrate of the FP sensor resulted in echoes in the time domain signal that could be removed by back filtering. The substrate acted as a filter in the frequency domain. The finite lateral sensor dimensions produced side waves that could be eliminated by surface averaging using an interrogation beam with adequate diameter. The fabricated FP sensor produced a noise-equivalent pressure = 0.76 kPa, bandwidth of 16.6 MHz, a spectral full-width at-half-maximum = 0.2886 nm, and quality factor Q=2694. Photoacoustic images were collected from phantoms and blood vessels in a live mouse.

**Conclusions:**

A miniature wafer-based FP sensor design has been demonstrated with scaled down form factor for future use in PAE.

## 1 Introduction

Photoacoustic (PA) imaging is an emerging technology that combines the use of light and sound to generate *in vivo* images with high spatial resolution and deep tissue penetration.^1^^–^^3^ Acoustic signal can be generated from endogenous contrast agents, such as hemoglobin (λ=532  nm), after absorbing energy from short laser pulses.^4^^,^^5^ A photoacoustic endoscope (PAE) can achieve superior imaging performance by providing greater access and direct contact with target tissues in internal organs, and by bypassing skin, a highly scattering medium. A sensor with form factor on the millimeter scale is needed for packaging in the distal tip of the instrument to detect acoustic signals with high sensitivity. There have been a number of previous demonstrations of PAE imaging systems. Opaque ultrasound transducers (UTs) based on piezoelectric materials are the most common detectors found in PAE systems.^4^^–^^10^ Most of these prototypes used a noncoaxial configuration^4^^–^^7^ to compensate for the opaque nature of the transducer, thus decreasing detection efficiency. Ring-shaped transducers^8^^–^^10^ have also been developed to form coaxial designs to address this challenge and often lead to an increase in instrument dimensions and fabrication complexity.

Fabry–Perot (FP) sensors provide a highly sensitive approach to detect acoustic waves and can be used for PAE if the detector dimensions can be scaled down sufficiently in size. The sensor design is based on the detection of reflected light from acoustically induced changes in the thickness of a polymer spacer. The sides consist of highly reflective mirrors to form an interferometer. Compared to piezoelectric transducers, FP sensors have the advantages in high sensitivity, broad bandwidth, small element size, and resistance to electromagnetic noises.^11^^–^^13^ Currently, the most common FP sensors used in PAE systems are fiber-based single-element sensors.^14^^–^^16^ Fiber bundle-based FP sensor arrays^17^^,^^18^ have been proposed, but high cost and limited array dimensions limit use. Large planar FP sensor arrays with thick substrates have also been demonstrated but are large in dimension.^12^^,^^13^ Previously, the transduction mechanisms of a planar sensor array with infinitely wide lateral size and semi-infinitely thick substrate have been modeled.^12^^,^^19^ Cox and Beard^20^ characterized the frequency-dependent directivity of a planar FP sensor. Buchmann et al.^21^ demonstrated an FP sensor with hard dielectric mirrors. Marques et al. have described the optical response of the FP sensor under different conditions, such as focused beam,^22^ arbitrary beam,^23^ and nonparallelism.^24^

A thin substrate-based planar miniature FP sensor would combine many of the advantages of the above designs. The thickness and lateral dimensions of a miniature FP sensor for PAE cannot be considered infinite, thus a mechanistic description of the performance of a finite thickness FP sensor is needed. Here we aim to demonstrate a detailed model to characterize the imaging performance of a wafer-based miniature FP polymer film acoustic sensor. Numerical and analytical models will be developed to determine the effects of finite thickness and lateral dimensions on imaging performance. Miniature sensors with dimensions of $2\times 2\;{\mathrm{mm}}^{2}$ will be fabricated using a parylene C polymer film, gold reflective mirrors, and glass wafer substrate. The sensitivity across the surface of the wafer will be characterized using a fixed wavelength laser. A back filtering method will be developed to eliminate echo artifacts introduced by a finite thickness substrate. Elimination of side waves will be demonstrated by increasing the diameter of the interrogation beam. Imaging phantoms will be used to verify sensor performance, and *in vivo* images of blood vessels from a live mouse will be demonstrated.

## 2 Methods

### 2.1 Transfer Matrix Model

A mathematical model was developed to characterize the frequency response of the miniature FP sensor to an incident pressure wave. A transfer matrix was generated to match the boundary conditions of pressure waves on either side of each interface. Matrix calculations were performed to determine the change in thickness of the parylene C film. Custom software was developed using MATLAB (MathWorks, Inc.) to generate model results. The transfer matrix was looped through different layers so that the model can be applied to structures with an arbitrary number of layers, including the simplest case where only tissue (water), parylene C film, and an infinitely thick glass layer were used. The model setup is shown in Fig. S1(a) in the Supplementary Material, and a detailed derivation has been provided in the Supplementary Material.

### 2.2 Analytical Model

An analytical model was developed to explicitly describe the effect of a substrate with finite thickness on the miniature FP sensor. This model follows the actual propagation of the incident pressure wave inside the sensor. The pressure wave incident on the parylene C film induces a change in thickness that is transmitted into the glass substrate and is reflected at the glass–air interface and travels back into the parylene C film to induce a secondary thickness change. This pressure wave is back reflected again at the glass–paryelene C interface, and reflections occur repeatedly inside the glass substrate to form an echo. The model setup is shown in Fig. S1(b) in the Supplementary Material and a detailed derivation is provided in the Supplementary Material.

### 2.3 Analysis for Finite Lateral Dimensions

For an FP sensor with finite lateral dimensions, a 2D finite-element analysis (FEA) model was developed using COMSOL Multiphysics (COMSOL Inc.) software to characterize the temporal response. The sensor consists of a parylene C film and glass substrate with a lateral width of 2 mm located below a $4\times 2\;{\mathrm{mm}}^{2}$ layer of tissue (modeled by water) above a layer of air with a depth of 0.1 mm. The change in sensor thickness was defined by the difference in the axial displacement at the center of the parylene C–tissue interface with that at the parylene C–glass interface. Infinitely wide sensors with the same parylene C and glass thicknesses were similarly modeled, except that film movement was constrained in the horizontal direction. The standard deviation of the resulting normalized data after the time domain signal of the infinitely wide sensor was subtracted from that of the finite sensor was used to quantify the effects of the finite lateral dimensions.

### 2.4 Design and Fabrication of Miniature FP Sensors

Gold films were used as reflective mirrors to design and fabricate the miniature FP sensors as a proof of concept with a helium–neon (He–Ne) interrogation laser. The second mirror was chosen as a 100 nm gold film to form a highly reflective mirror. The optimized thickness of the first mirror was found to be 32.8 nm by calculating the sensitivity of the FP sensor resulting from the corresponding interferometer transfer function (ITF).

$10\times 10\;{\mathrm{mm}}^{2}$ miniature FP sensors were fabricated. 10 Å titanium (Ti), 32.8 nm gold (Au), and another 10 Å Ti were magnetron sputtered (Kurt J Lesker Company, #Lab 18-2) successively on a borosilicate glass substrate with 100 mm diameter and 500  μm thickness. Next, 32  μm parylene-C was vacuum deposited (Specialty Coating Systems Inc., #PDS 2035) on the Ti layer to achieve a −3  dB bandwidth of ∼20  MHz. A 10 Å layer of Ti and a 100 nm layer of Au were then sputtered. The wafer was spin coated (Brewer Science #CEE 200X) with a 3.2  μm layer of photoresist (Dow Inc., #SPR 220 3.0), and the wafer was partially diced with dimensions of $10\times 10\;{\mathrm{mm}}^{2}$ with a 200  μm thick substrate remaining for characterization (Advanced Dicing Technologies Ltd., #ADT 7100 Dicing Saw).

Single-element sensors were fabricated, as described above, by dicing the wafer along trenches into $2\times 2\;{\mathrm{mm}}^{2}$ sensors. The wafer was first broken into $10\times 10\;{\mathrm{mm}}^{2}$ square pieces along the trenches for characterization. The $2\times 2\;{\mathrm{mm}}^{2}$ regions with relatively uniform sensitivity were identified for use as single-element FP sensors. The sensors were cleaned with acetone and isopropyl alcohol successively to remove the photoresist and deposited with a 4  μm layer of parylene C as a protection layer.

### 2.5 Sensor Characterization

#### 2.5.1 $10\times 10\;{\mathrm{mm}}^{2}$ sensor

A tabletop imaging system was built to characterize the sensitivity patterns of the $10\times 10\;{\mathrm{mm}}^{2}$ FP sensors [Fig. S3(a) in the Supplementary Material]. The sensor was mounted in the side wall of a water tank with the Au-coated side facing water. A cw He–Ne laser (REO, #30990, λ=633  nm, 5 mW) was expanded by a pair of lenses $L_{1}$ (f=18.4  mm at 780 nm, Thorlabs, #C280TME-A) and $L_{2}$ (f=50  mm at 587.6 nm, Thorlabs, #LA1213-A). The expanded beam was focused by an achromatic lens $L_{3}$ (f=75  mm, Thorlabs, #AC508-075-A) onto the glass surface of the FP sensor. The sensor was placed at a small angle (∼4  deg) with respect to the incident beam to allow for the reflected beam to be incident on mirror $M_{5}$ (Thorlabs, #BBSQ05-E02). This beam was focused by lens $L_{4}$ (f=50  mm at 587.6 nm, Thorlabs, #LA1213-A) onto an ac-coupled low-noise photodetector (PD, New Focus, #1801). A linear stage with 3 dc servo actuators (Newport, #CONEX-TRA12CC) was used to raster scan the tank and sensor. A 10 MHz unfocused UT (Olympus, #V312-SU) controlled by a pulser-receiver (Olympus, #PR5073) was used to characterize the FP sensor surface sensitivity. A hydrophone (ONDA, #HNC-1500) was used to characterize the ultrasound wave generated by the transducer and calculate the noise-equivalent pressure (NEP). The sensor bandwidth was determined by ratioing the spectrum of the output signal with that of the input ultrasound wave.

The ITF of the sensor was characterized using an external cavity diode laser (New Focus, #TLB-6700 Velocity) with a wavelength range of 765 to 781 nm. This laser was used to estimate the actual performance of the sensor at 633 nm. The beam coming out of the laser was reflected by a sample sensor onto a power meter. The measured ITF was fitted with an asymmetric model^25^ and the full-width-at-half-maximum (FWHM) was measured.

#### 2.5.2 $2\times 2\;{\mathrm{mm}}^{2}$ sensor

A tabletop imaging system was modified to characterize the sensitivity patterns of the $2\times 2\;{\mathrm{mm}}^{2}$ FP sensors [Fig. S3(b) in the Supplementary Material]. A polarizer (Thorlabs, #LPNIRE100-B) was placed after the output of the He–Ne laser. A polarizing beam splitter (PBS, Thorlabs, #PBS122) and a quarter wave plate (QWP, Thorlabs, #WPQSM05-633) were used to transform the linearly polarized beam to a circularly polarized beam. The laser beam was reflected by a fixed mirror (Thorlabs, #PF10-03-P01), which was mounted on a kinematic mirror mount (Thorlabs, #KM100), downward onto the glass substrate of the sensor. The sensor was mounted on a 3D printed holder, which was controlled by a manual stage to align the center of the sensor with the laser beam and adjust the proper height. The reflected beam from the sensor was transformed back to a linearly polarized beam by the same QWP with a perpendicular polarization state with respect to the original beam. Finally, the beam directed by the PBS was filtered by a laser line filter (Thorlabs, #FL632.8-3) and detected by a low-noise PD (New Focus, #1801).

A custom assembly was used to hold the imaging target. First, the imaging target was placed on top of a block-shape holder placed on the bottom of the assembly. The holder height was designed to match that of the imaging target and container wall. The target was held down by a plastic tank that was placed on top of the bottom container. A plastic membrane was used to seal a square-shaped hole in the tank bottom to form an imaging window. The top tank was filled with water. The water, membrane, and the target achieved good acoustic coupling using US imaging gel (EcoVue, #286). The whole assembly was placed on a 2D motorized stage. The excitation subprobe illuminated the target through the membrane at an angle and was controlled using a manual stage. The excitation beam focus was ∼5.5  mm from the sensor. The whole container assembly was raster scanned in 2D to form 3D images. The A-line data at each pixel was Hilbert transformed to acquire the depth profile.

### 2.6 Elimination of Echo Signals Using Back Filtering

Back filtering was performed to eliminate the echo signals in the substrate. The measured layer properties of the miniature FP sensor were used to verify the back filter parameters. An optimization was performed using custom MATLAB software to identify the layer properties prior to back filtering. The program loops through the layer properties, and back filtering was performed using the original data measured. The main signal without echoes and side waves was extracted as the reference data. The reference data were then subtracted from the back-filtered data, and the standard deviation of normalized data was used to determine the merit function.

### 2.7 Subprobes for $2\times 2\;{\mathrm{mm}}^{2}$ Single-Element FP Sensors

Single-element sensors were fabricated, as described above, by dicing the wafer along trenches into $2\times 2\;{\mathrm{mm}}^{2}$ sensors. The wafer was first broken into $10\times 10\;{\mathrm{mm}}^{2}$ square pieces by hand along the trenches for characterization using the set-up mentioned above. The $2\times 2\;{\mathrm{mm}}^{2}$ regions with relatively uniform sensitivity were identified for use as single-element FP sensors.

Miniature subprobes were developed to deliver the excitation beam for use in PAE instruments. The subprobe consisted of a light delivery optical fiber, a fiber ferrule to center the fiber, a GRIN lens to focus or collimate the light coming out of the fiber, and a stainless-steel tube (SST) to contain the optical components.

An INNOSLAB $\mathrm{Nd}:{\mathrm{VO}}_{4}$ laser (EdgeWave, #BX60-2-G, $\lambda_{\mathrm{ex}}=532\;\mathrm{nm}$, <6.8  ns, 0.3 mJ at 100 kHz) was selected for use as the excitation source to image hemoglobin. A GRIN lens with a custom antireflection coating (Grintech, #GT-LFRL-100-025-50-C1) was used. To deliver as much pulse energy as possible without damaging the optics, a 105  μm core multimode fiber (MMF, Thorlabs, #FG105LVA) was used. To accurately model the propagation of light in an MMF, nonsequential simulation using Zemax software (version 22.2) was performed. The MMF was modeled as two concentric cylinders with different indices of refraction. The optical properties of the core and cladding and the input numerical aperture were obtained from the MMF spec sheet.

The MMF was glued to a common 125  μm inner diameter (ID) fiber ferrule. A special glass ferrule (VitroCom, #9235) with a 1.00 mm outer diameter (OD), 0.27 mm ID, and 5 mm length were used. The MMF was inserted through the ferrule, and cut by a fiber cleaver (Fujikura, #CT50). The MMF was then glued at the other end of the ferrule with ultraviolet (UV) glue (Norland Products Inc., #NOA 61).

An SST (McMaster, #5560K46) with 1.27 mm (0.05 in) OD, 0.1016 mm (0.004 in) wall thickness, and 1.07 mm (0.042 in) ID were used. The upper part of the SST was removed using a custom-made abrasive machining tool to better monitor the positions of the components during assembly. The GRIN lens was first put into the SST manually and fixed using UV glue. The glass ferrule was mounted on a manual stage and was carefully positioned using two microscopes that provided either a top or side view. The ferrule was placed into the SST using a manual stage. The distance between the fiber tip and the GRIN lens was determined by monitoring the output diameter of the beam at an imaging depth of 5.5 mm. The beam diameter was measured using a beam profiler (Thorlabs, #BP209-VIS). The ferrule was fixed to the SST using UV glue after the desired beam diameter was achieved.

A second subprobe was built using a 25  μm core MMF (Thorlabs, #FG025LJA) to provide higher fluence. A nonsequential Zemax simulation was performed to find the optimal distance between the fiber tip and GRIN lens. The same assembly process, as described above, was used to fabricate the subprobe.

### 2.8 Phantom Imaging Using Subprobes

Custom phantoms were used to verify the performance of the imaging system. A set of three pencil leads (300  μm diameter) in Figs. S6(a)–S6(c) in the Supplementary Material and three (42 AWG) magnet wires (63  μm diameter) in Figs. S6(d)–S6(f) in the Supplementary Material were placed in a horizontal plane with a separation of 1 mm. The phantoms were embedded in PDMS to mimic tissue scattering properties.

### 2.9 In Vivo Imaging of Nude Mouse Ear

Imaging studies were performed with approval of the University of Michigan Committee on the Use and Care of Animals. A nude mouse (NU/J, 002019, The Jackson Lab) was used to collect photoacoustic images from blood vessels in the ear. The nude mouse was placed in the bottom container of the assembly [Fig. S3(b) in the Supplementary Material]. The ear was first administered hair remover and then rinsed with distilled water. Next US gel was applied to the ear prior to placing the top tank on the ear. The ear was placed in good contact with the membrane of the imaging window.

## 3 Results

### 3.1 Transfer Matrix Model

The transmitted pressure wave out of the glass substrate into air $P_{T}$ can be expressed by the input pressure wave $P_{0}$ at the parylene C–tissue interface and the reflected pressure wave $P_{R}$ from this interface as described by the transfer matrix model [Fig. S1(a) in the Supplementary Material]:

$$[\begin{array}{c} P_{T}\\ 0 \end{array}]={\left[\begin{array}{cc} e^{-ik_{4}\left(l+H\right)} & e^{ik_{4}\left(l+H\right)}\\ \frac{1}{z_{4}}e^{-ik_{4}\left(l+H\right)} & -\frac{1}{z_{4}}e^{ik_{4}\left(l+H\right)} \end{array}\right]}^{-1}\times [\begin{array}{cc} e^{-ik_{3}\left(l+H\right)} & e^{ik_{3}\left(l+H\right)}\\ \frac{1}{z_{3}}e^{-ik_{3}\left(l+H\right)} & -\frac{1}{z_{3}}e^{ik_{3}\left(l+H\right)} \end{array}]\times {\left[\begin{array}{cc} e^{-ik_{3}l} & e^{ik_{3}l}\\ \frac{1}{z_{3}}e^{-ik_{3}l} & -\frac{1}{z_{3}}e^{ik_{3}l} \end{array}\right]}^{-1}\times [\begin{array}{cc} e^{-ik_{2}l} & e^{ik_{2}l}\\ \frac{1}{z_{2}}e^{-ik_{2}l} & -\frac{1}{z_{2}}e^{ik_{2}l} \end{array}]\times {\left[\begin{array}{cc} 1 & 1\\ \frac{1}{z_{2}} & -\frac{1}{z_{2}} \end{array}\right]}^{-1}\times [\begin{array}{cc} 1 & 1\\ \frac{1}{z_{1}} & -\frac{1}{z_{1}} \end{array}]\times [\begin{array}{c} P_{0}\\ P_{R} \end{array}]=[\begin{array}{cc} M_{11} & M_{12}\\ M_{21} & M_{22} \end{array}][\begin{array}{c} P_{0}\\ P_{R} \end{array}]=M[\begin{array}{c} P_{0}\\ P_{R} \end{array}],$$ (1)

where $M=[\begin{array}{cc} M_{11} & M_{12}\\ M_{21} & M_{22} \end{array}]$ is defined as the system transfer matrix. $k_{1}$, $k_{2}$, $k_{3}$, and $k_{4}$ are the wave numbers for pressure waves in tissue, parylene C, glass, and air, respectively. $k_{1}=\frac{\omega }{c_{1}}$, $k_{2}=\frac{\omega }{c_{2}}$, $k_{3}=\frac{\omega }{c_{3}}$, $k_{4}=\frac{\omega }{c_{4}}$, where ω is the angular frequency of the wave, $c_{1}$, $c_{2}$, $c_{3}$, and $c_{4}$ are the speeds of sound in tissue, parylene C, glass, and air. $z_{1}$, $z_{2}$, $z_{3}$, and $z_{4}$ are the acoustic impedances in tissue, parylene C, glass, and air, respectively. $z_{n}=\rho_{n}c_{n}$, where $\rho_{n}$ is the density of the material and the $c_{n}$ is the corresponding speed of sound. The thickness of the parylene C film is l, and the thickness of the glass substrate is defined as H.

We have $0=M_{21}P_{0}+M_{22}P_{R}$, which gives $P_{R}=-\frac{M_{21}}{M_{22}}P_{0}$. Then $P_{1}$ and $P_{2}$, which represent the pressure waves travelling in the positive and negative X directions inside of the parylene C film, can be represented as

$$[\begin{array}{c} P_{1}\\ P_{2} \end{array}]={\left[\begin{array}{cc} 1 & 1\\ \frac{1}{z_{2}} & -\frac{1}{z_{2}} \end{array}\right]}^{-1}[\begin{array}{cc} 1 & 1\\ \frac{1}{z_{1}} & -\frac{1}{z_{1}} \end{array}][\begin{array}{c} P_{0}\\ P_{R} \end{array}],$$ (2)

$$P_{1}=\frac{1}{2}[(1+\frac{z_{2}}{z_{1}})P_{0}+(1-\frac{z_{2}}{z_{1}})P_{R}]=\frac{1}{2}[(1+\frac{z_{2}}{z_{1}})-\frac{M_{21}}{M_{22}}(1-\frac{z_{2}}{z_{1}})]P_{0},$$ (3)

$$P_{2}=\frac{1}{2}[(1-\frac{z_{2}}{z_{1}})P_{0}+(1+\frac{z_{2}}{z_{1}})P_{R}]=\frac{1}{2}[(1-\frac{z_{2}}{z_{1}})-\frac{M_{21}}{M_{22}}(1+\frac{z_{2}}{z_{1}})]P_{0}.$$ (4)

Finally, the change in thickness of the parylene C film can be represented as

$$\text{Δ}l(\omega )=\frac{\left(P_{1}e^{-ik_{2}l}-P_{2}e^{ik_{2}l})-(P_{1}-P_{2}\right)}{i\omega z_{2}}=\frac{e^{-ik_{2}l}-1}{i\omega z_{2}}(P_{1}+P_{2}e^{ik_{2}l})=\frac{e^{-ik_{2}l}-1}{2i\omega z_{2}}\{[(1+\frac{z_{2}}{z_{1}})-\frac{M_{21}}{M_{22}}(1-\frac{z_{2}}{z_{1}})]+e^{ik_{2}l}[(1-\frac{z_{2}}{z_{1}})-\frac{M_{21}}{M_{22}}(1+\frac{z_{2}}{z_{1}})]\}P_{0}=F(\omega )P_{0}(\omega ),$$ (5)

where F(ω) is the frequency response of the multiple layer sensor. In the frequency domain, the induced thickness change was simply the frequency response of the sensor multiplied by the spectrum of the input signal.

**Fig. 1:**
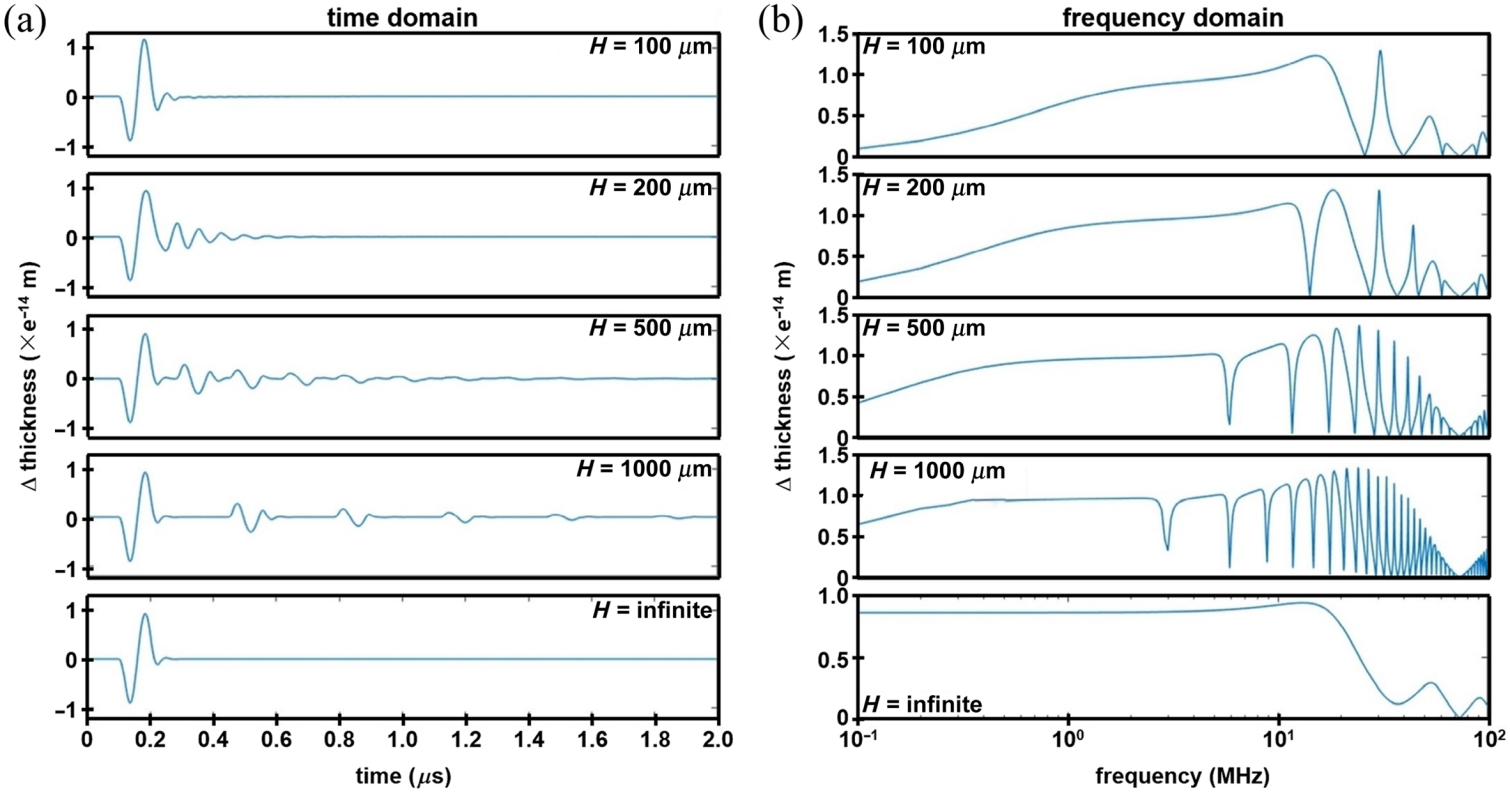
Transfer matrix results. The normalized change in parylene C film thickness is shown from an incident pressure wave $P_{0}$ in the (a) time and (b) frequency domains for a miniature FP sensor with a glass substrate that varies in thickness H from 100 to 1000  μm and infinite.

Simulation results in the time and frequency domains using the transfer matrix model are shown in Figs. 1(a) and 1(b). The normalized change in parylene C thickness and corresponding frequency response are shown for the glass substrate with thicknesses that vary from 100 to 1000  μm and infinite. The results for the infinite-thick sensor agreed with the results calculated using the proposed method.^12^ The thickness of the parylene C film was chosen to be 32  μm to provide a −3  dB bandwidth of about 20 MHz for the infinitely thick sensor. The same parylene C thickness was used for the other sensors with finite glass thicknesses. The frequency response of the sensors with finite thicknesses exhibited quite different patterns compared with that for infinitely thick sensors and showed no well-defined bandwidth. These results indicated that the frequency response of a miniature sensor differed from that predicted by previous models, thus requiring more consideration in conducting experiments, signal processing, and image reconstruction. These patterns resulted from the fact that the pressure wave inside the substrate undergoes multiple reflections and form echoes. The echoes can be observed in the time domain. The time interval between echoes was equal to the time of flight for the pressure wave to travel a round trip inside the substrate, i.e., $\Delta t=2H/c_{3}$. As H increases, the echoes moved further away from the main signal induced by the incident pressure wave $P_{0}$. As H decreased, the echoes merged into the main signal as the glass layer becomes acoustically thin. The echoes introduced an undesired contribution to the data, resulting in artifacts in the photoacoustic images.

### 3.2 Analytical Model

An analytical model was developed to describe the pressure waves undergoing multiple reflections inside the sensor substrate [Fig. S1(b) in the Supplementary Material]. The total pressure $P_{G}$ inside of the glass was the sum of all the reflected pressure waves and can be calculated as follows:

$$P_{G}=P^{\left(1\right)}+P^{\left(2\right)}+P^{\left(3\right)}\ldots =P^{\left(1\right)}+P^{\left(1\right)}R_{1}R_{2}+P^{\left(1\right)}{\left(R_{1}R_{2}\right)}^{2}+\ldots =P^{\left(1\right)}\overset{\infty }{\underset{n=0}{\sum }}{\left(R_{1}R_{2}\right)}^{n}\;=P^{\left(1\right)}\frac{1}{1-R_{1}R_{2}}=P_{0}TR_{1}\frac{1}{1-R_{1}R_{2}},$$ (6)

where $P^{\left(1\right)},P^{\left(2\right)},P^{\left(3\right)}\ldots$ are the pressure waves that arrive at the parylene C–glass interface after the first, second, third … reflections by the glass–air interface. $P_{0}$ is the incident pressure wave on the parylene C film. $R_{1}$ is the reflection coefficient between air and an infinitely thick glass substrate. $R_{2}$ is the reflection coefficient between an infinite glass substrate and a parylene C film with thickness l. T is the acoustic transmission coefficient through the parylene C film. The detailed expressions of these complex parameters are shown in the Supplementary Material.

The total thickness change of a sensor with a glass substrate Δl can be written as the sum of the thickness changes $\Delta l_{0}$ induced by $P_{0}$ and $\Delta l_{G}$ induced by $P_{G}$:

$$\Delta l=\Delta l_{0}+\Delta l_{G}.$$ (7)

Since $\Delta l_{0}$ is proportional to $P_{0}$ and $\Delta l_{G}$ is proportional to $P_{G}$, Δl can be calculated as

$$\Delta l=\Delta l_{0}+\Delta l_{0}TR_{1}\eta \frac{1}{1-R_{1}R_{2}}=\Delta l_{0}(1+\frac{TR_{1}\eta }{1-R_{1}R_{2}}),$$ (8)

where η is defined as the ratio between the change in thickness of the parylene C film caused by individual and equal incident waves from infinite-thick glass and tissue sides as a result of different boundary conditions at each interface. Thus Δl can be written as $\Delta l(\omega )=F_{H}(\omega )\Delta l_{0}(\omega )$, where $F_{H}(\omega )=1+\frac{TR_{1}\eta }{1-R_{1}R_{2}}$ is defined as a frequency domain filter that converts the original sensor frequency response $\Delta l_{0}(\omega )$ to that of the new finite thickness sensor Δl(ω). After including expressions for the coefficients and simplifying the result, the explicit expression for $F_{H}(\omega )$ is

$$F_{H}(\omega )=\frac{1-e^{-2ik_{3}H}\frac{z_{3}\;\cos(\frac{1}{2}k_{2}l)-iz_{2}\;\sin(\frac{1}{2}k_{2}l)}{z_{3}\;\cos(\frac{1}{2}k_{2}l)+iz_{2}\;\sin(\frac{1}{2}k_{2}l)}}{1-e^{-2ik_{3}H}\frac{\left(1-\frac{z_{1}}{z_{3}})\cos(k_{2}l)+i(-\frac{z_{2}}{z_{3}}+\frac{z_{1}}{z_{2}})\sin(k_{2}l\right)}{\left(1+\frac{z_{1}}{z_{3}})\cos(k_{2}l)+i(\frac{z_{2}}{z_{3}}+\frac{z_{1}}{z_{2}})\sin(k_{2}l\right)}}.$$ (9)

The frequency response of a finite thickness sensor is simply that of an infinitely thick sensor multiplied by $F_{H}(\omega )$.

**Fig. 2:**
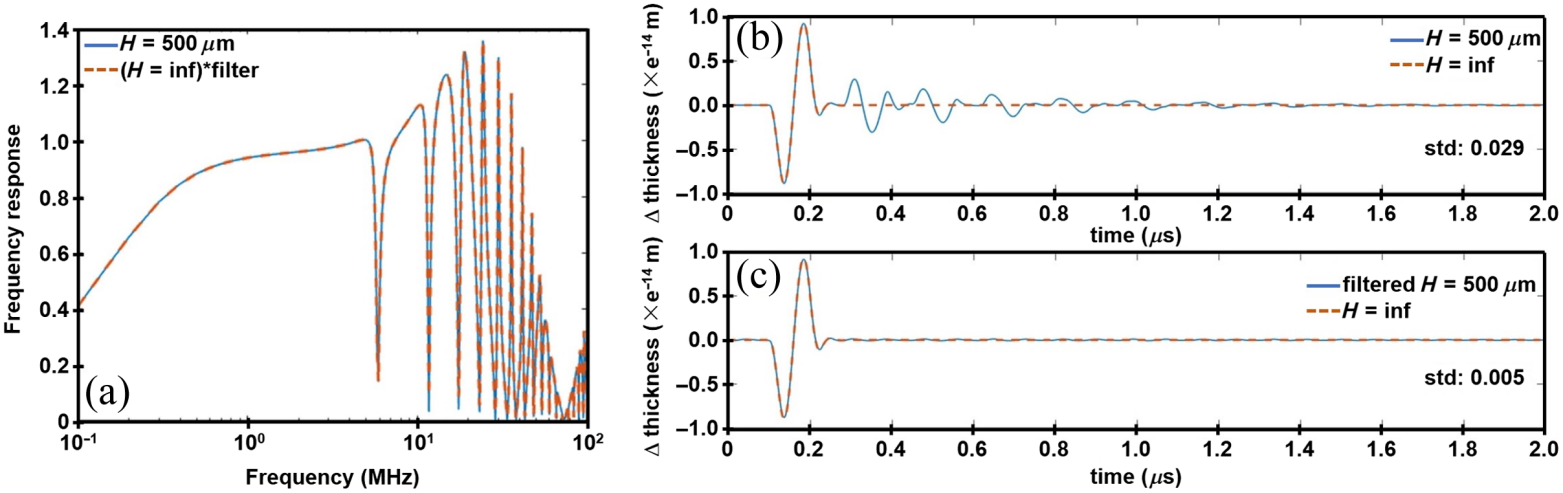
Frequency response. (a) Results for miniature FP sensor with 500  μm thick glass substrate (blue) are similar to that for an infinitely thick substrate (orange) after filtering. (b) Time domain signals for sensor with 500  μm thick glass substrate show presence of echoes (c) that are removed after filtering. The difference in signal from H = inf before and after filtering has a standard deviation of 0.029 and 0.005, respectively.

Simulation results using Eq. (9) are shown for the frequency response of an FP sensor with an H=500  μm glass substrate. These results are compared with the product of the frequency response for an infinitely thick sensor (H=inf) and the frequency domain filter $F_{H}(\omega )$ [Fig. 2(a)]. In the time domain, the response from a 500  μm and an infinitely thick sensor are shown in Fig. 2(b). Filtering can remove the echoes introduced in the substrate [Fig. 2(c)]. The difference in signal between the finite and infinite sensor showed a standard deviation after filtering that decreased from 0.029 in to 0.005.

### 3.3 Analysis for Finite Lateral Dimensions

**Fig. 3:**
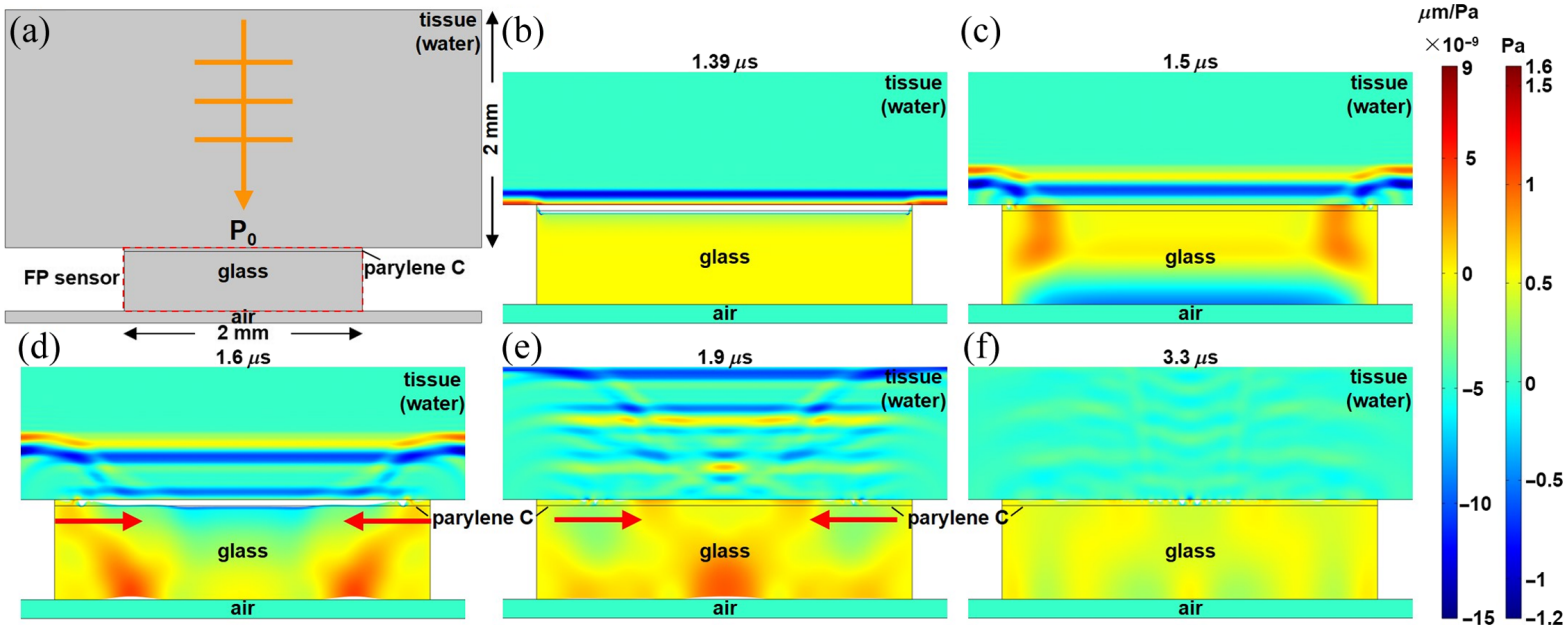
Finite sensor dimensions. Unit pressure wave $P_{0}$ is incident on a 2 mm wide FP sensor (32  μm parylene C film and 500  μm glass substrate) after passing through a 2 mm deep tissue (water) domain adjacent to air (0.1 mm). (a) Model geometry is shown. Acoustics wave propagation and sensor deformation are shown at various time points, including (b) 1.39, (c) 1.5, (d) 1.6, (e) 1.9, and (f) 3.3  μs. Left color bar: the vertical displacement inside of the parylene film and glass substrate per unit incident pressure ($\times 10^{-9}\;\mu m/\mathrm{Pa}$). Right color bar: the pressure inside of the tissue (water).

Simulations were performed to characterize the pressure waves in a miniature FP sensor with finite lateral dimensions. A 2 mm layer of tissue was modeled using the acoustic properties of water [Fig. 3(a)]. A 32  μm parylene C film was used to provide a −3  dB bandwidth of 20 MHz. A 200  μm glass substrate was used so that the echoes caused by the substrate were close to the main signal and did not interfere with the waves caused by the finite lateral dimensions. The results showed that the initial change in the thickness of the parylene C film occurs at ∼1.39  μs after arrival of the incident pressure wave $P_{0}$ [Fig. 3(b)]. The pressure wave experienced multiple reflections inside the glass substrate. The fixed edges of the parylene C film resulted in lateral deformations [Fig. 3(c)]. The deformation propagated (red arrows) as side waves that traveled toward the center [Figs. 3(d) and 3(e)]. The side waves met at the center at ∼3.3  μs [Fig. 3(f)] and occurred when the thickness change at the center caused by the side waves reached maximum amplitude. The side waves kept propagating and gradually disappeared.

**Fig. 4:**
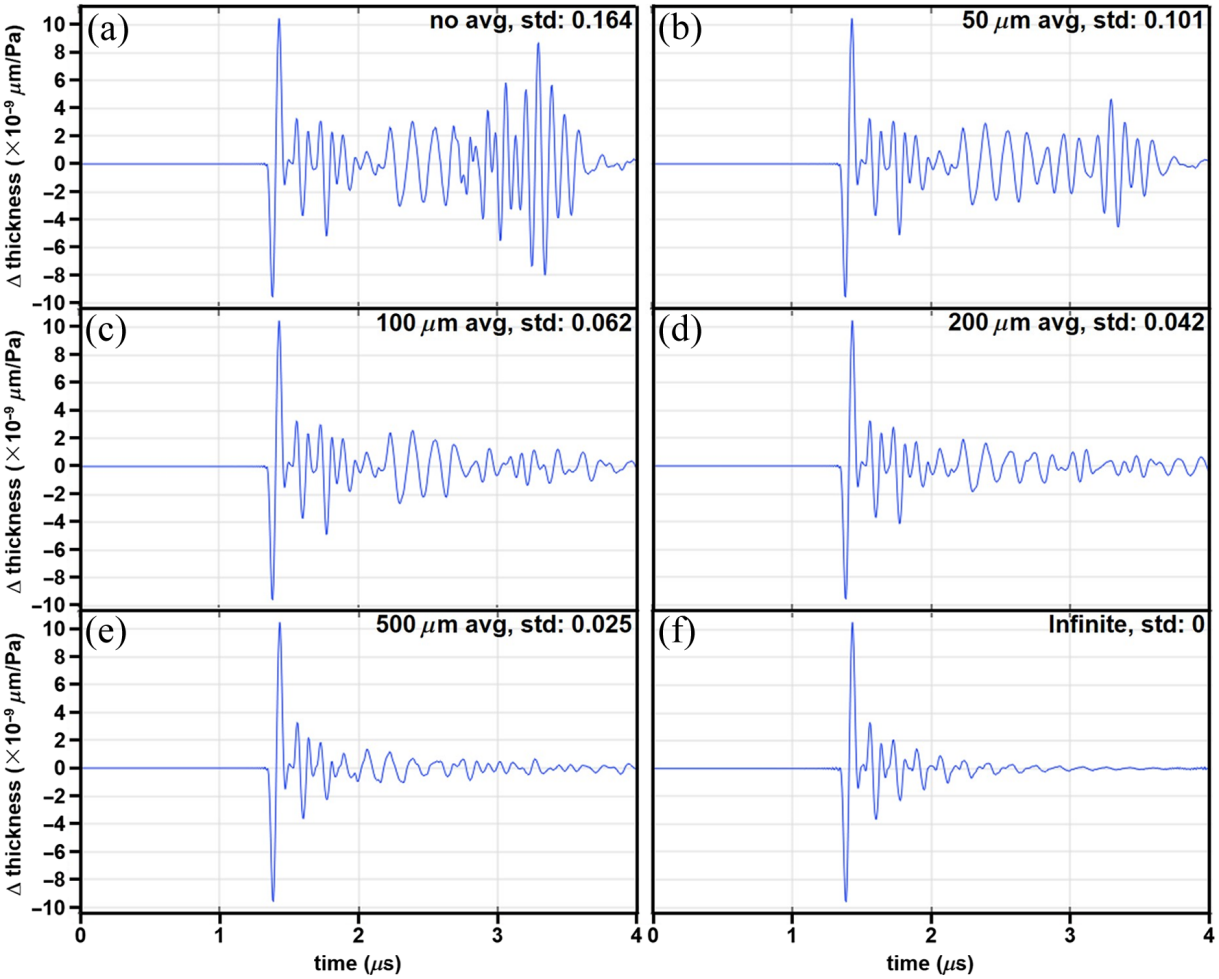
Interrogation beam dimensions. The change in thickness ($\times 10^{-9}\mu m/\mathrm{Pa}$) is shown for a 2 mm wide FP sensor (32  μm thick parylene C film and 0.5 mm thick glass substrate) from an incident pressure wave $P_{0}$. Different averaging lengths, including (a) 0, (b) 50, (c) 100, (d) 200, and (e) 500  μm, and (f) infinite, result from increasing the dimensions of the interrogation beam. The difference in signal from H = infinite before and after filtering has a standard deviation that decreases from 0.164 (no averaging) to 0.025 with a 500  μm averaging length.

The sensor was illuminated by an interrogation laser with a finite beam diameter, and the total signal measured resulted from the change in thickness of the parylene C film averaged over the beam dimensions. Thus the beam spot size affects the side waves generated in the parylene C film. Since the simulation was done in 2D, a line average method was used to simulate the averaging effect over the circular beam dimensions. The change in thickness for the parylene C film for a sensor with 2 mm lateral length, 0.5 mm glass substrate thickness, and 32  μm parylene C film thickness using an averaging length of 0, 50, 100, 200, and 500  μm are shown in Figs. 4(a)–4(e). The response of an infinitely wide sensor is shown in Fig. 4(f). Without averaging, the magnitude of the side waves was quite significant compared to the main signal, producing a standard deviation of the normalized differential signal of 0.164. However, the standard deviation was substantially reduced using a 100 and 200  μm averaging length down to 0.062 and 0.042, respectively, and was further decreased to 0.025 using a 500  μm length. Simulation results for variations in thickness of the glass substrate, lateral size of the sensor, and thickness of the parylene C film are provided in the Supplementary Material and Fig. S4 in the Supplementary Material.

### 3.4 Sensor Characterization

**Fig. 5:**
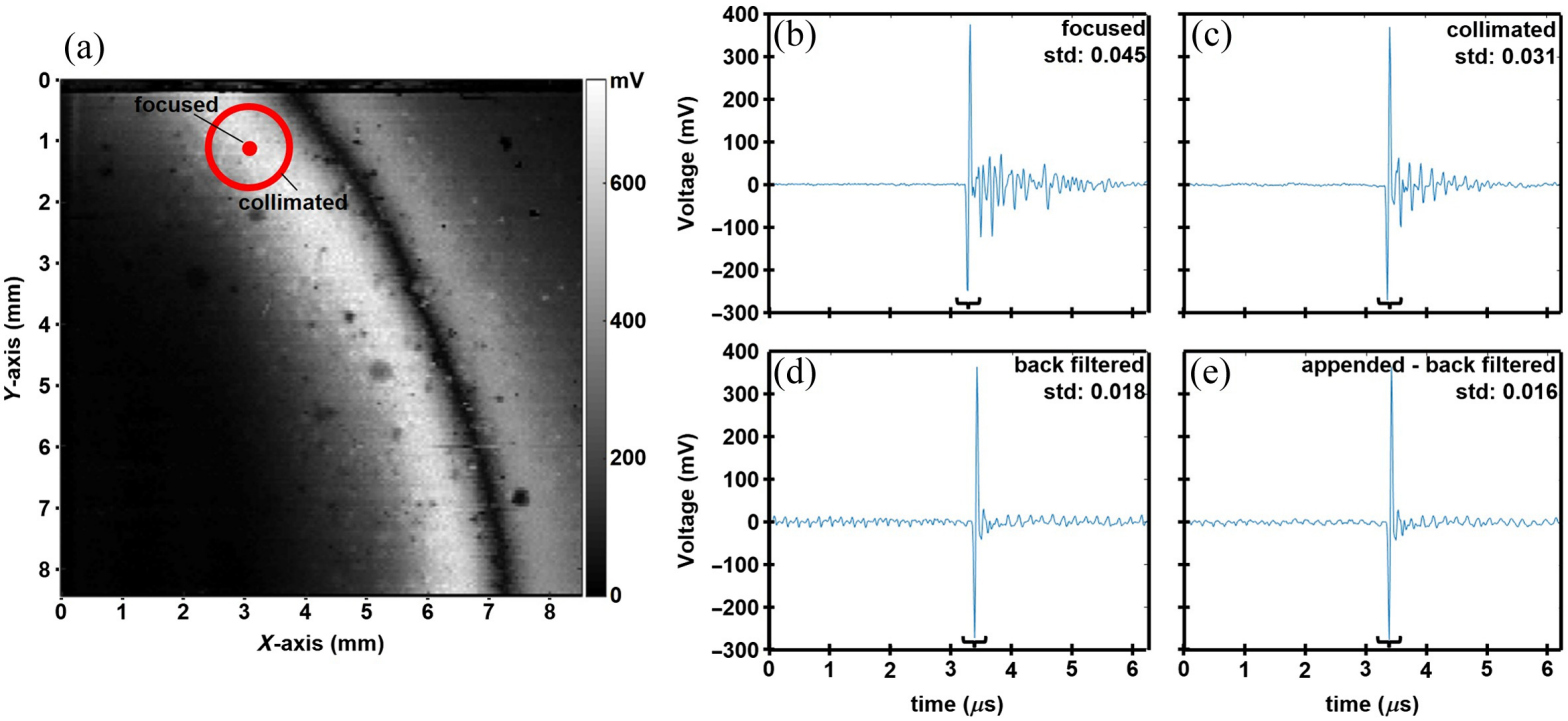
Time domain signals and back filtering. (a) Surface sensitivity map is shown for a representative $10\times 10\;{\mathrm{mm}}^{2}$ FP sensor. Time domain signals were acquired with a (b) focused and (c) collimated laser beam with diameters of 121  μm and 1.5 mm, respectively. The standard deviations after subtracting out the main signal are 0.045 and 0.031, respectively. The back filtered time domain signal is shown for the (d) original and (e) appended signal from (c). The standard deviations are reduced to 0.018 and 0.016, respectively.

A photo of $10\times 10\;{\mathrm{mm}}^{2}$ miniature FP sensors diced from a 100 mm diameter wafer is shown in Fig. S2(a) in the Supplementary Material. The sensitivity maps of individual sensors were measured and arranged in Fig. S2(b) in the Supplementary Material. The fringes resulted from the variations in parylene C thickness across the wafer. A sensitivity map for a representative $10\times 10\;{\mathrm{mm}}^{2}$ FP sensor is shown in Fig. 5(a). A 121  μm focused beam was used to interrogate the sensor. The peak-to-peak voltage of the time domain signal was used to reflect the sensitivity at each location. The time domain signal is shown at a representative location (red dot) acquired with a focused beam [Fig. 5(b)]. The main signal from the incident pressure wave is followed by a superposition of echoes and side waves from the glass substrate and the finite lateral dimensions, respectively. The main signal is identified (black bracket). The standard deviation of the signal after subtracting the main signal was 0.045. Next, a 1.5 mm diameter collimated beam was used to interrogate the sensor (red circle). The center coincided with the representative focus spot. The reflected beam was collected to produce the time domain signal [Fig. 5(c)]. Compared with Fig. 5(b), periodical echoes appeared after the main signal, indicating the effect of the finite substrate. The standard deviation after subtracting out the main signal was 0.031, thus the effect of finite lateral size was substantially reduced. The best NEP was 0.76 kPa, and the average NEP was 1.12 kPa in the most sensitive region.

**Fig. 6:**
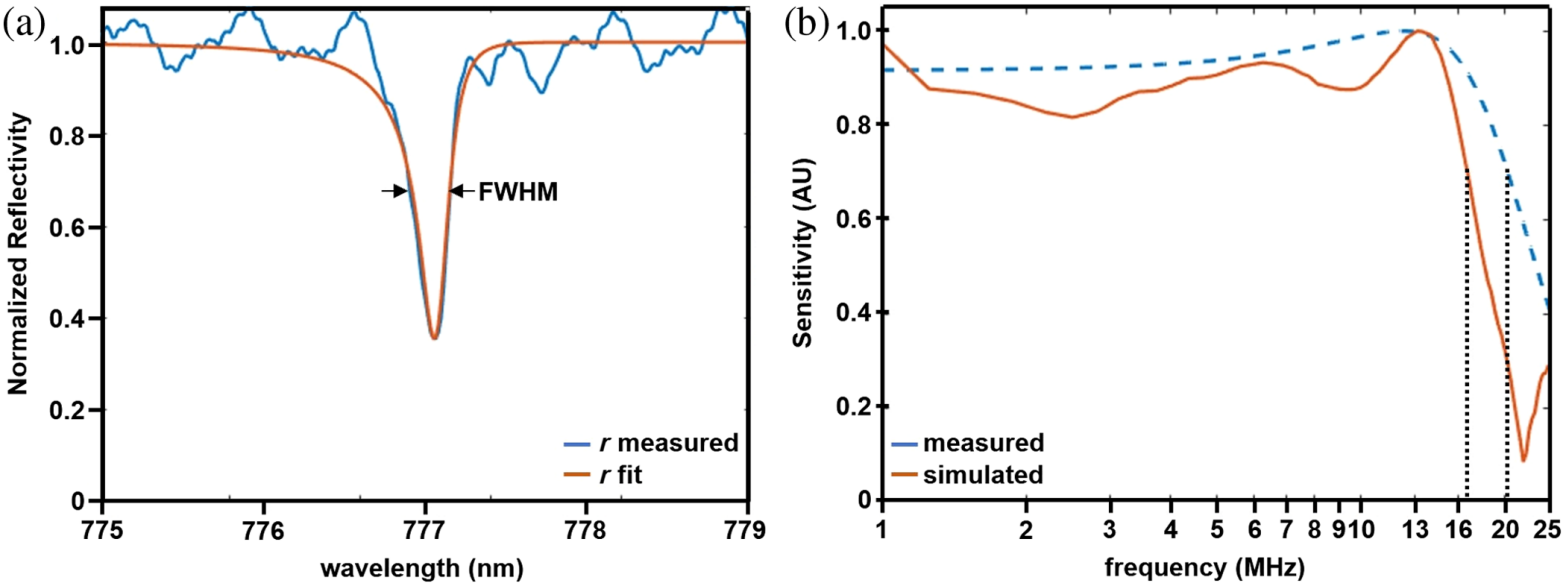
ITF and sensor bandwidth. (a) The normalized reflectivity of the miniature FP sensor showed an FWHM = 0.29 nm and a quality factor Q=2694 over the range of 775 to 779 nm. (b) The measured (blue) and (orange) simulated frequency responses showed a −3  dB bandwidth (dotted lines) of 16.6 and 20.3 MHz, respectively.

The normalized reflectivity of the FP sensor measured in the range of 775 to 779 nm is shown in Fig. 6(a). The FWHM measured from fitting the curve based on an asymmetric model^25^ was 0.29 nm, resulting in a quality factor of 2694. The frequency response is shown in Fig. 6(b), and a −3  dB bandwidth of 16.6 MHz was measured from the plot.

### 3.5 Elimination of Echo Signals Using Back Filtering

The optimal back filter properties were found with parameters of l=23.3  μm, $\rho_{2}=1142\;\mathrm{kg}/m^{3}$, $c_{2}=2549\;m/s$, $\rho_{3}=3126\;\mathrm{kg}/m^{3}$, and $c_{3}=5499\;m/s$, with $\rho_{2}$ and $c_{2}$ being the density and speed of sound in the parylene C film, and $\rho_{3}$ and $c_{3}$ being the density and speed of sound in the glass substrate. The Fourier transform of the original data was divided by the filter calculated using these optimal properties, and the results were inversely Fourier transformed back into time domain. The back-filtered data are shown in Fig. 5(d). The standard deviation of the signal after subtracting out the main signal was 0.018, which decreased from 0.031 [Fig. 5(c)], resulting in a substantial reduction of echoes from multiple reflections of pressure waves inside the substrate. The same back filtering operation was performed, and the results are shown in Fig. 5(e). The artifacts in the signals were further decreased, with the standard deviation after subtracting out the main signal was reduced to 0.016.

### 3.6 Subprobes for $2\times 2\;{\mathrm{mm}}^{2}$ Single-Element FP Sensors

Ray trace simulations were performed to characterize beam propagation inside the subprobes used to deliver laser pulses to excite photoacoustic signal for detection by the $2\times 2\;{\mathrm{mm}}^{2}$ single-element FP sensors. Results for the 105  μm core MMF are shown in Fig. S5(a) in the Supplementary Material. An optimal distance of 0.158 mm between the MMF and the GRIN lens produced a 664  μm diameter beam spot at the imaging plane. A beam diameter of 1 mm was used to prevent the high fluence of the excitation pulses from damaging the optics, resulting in a distance between the MMF and GRIN lens of 0.8 mm. An energy output of 79  μJ was measured at a control current of 40 A, and the coupling efficiency was 85%. A photo of the assembled subprobe is shown in Fig. S5(c) in the Supplementary Material.

A second subprobe was developed using a 25  μm diameter MMF. The nonsequential ray trace simulation showed an optimal distance of 0.13 mm between the fiber tip and GRIN lens, resulting in a beam diameter of 152.5  μm at a 5.5 mm imaging depth. However, this distance was increased to 0.38 mm to decrease the fluence at the GRIN lens surface. A beam diameter of 350  μm was measured, and simulation results for this separation are shown in Fig. S5(b) in the Supplementary Material. The predicted beam diameter at a depth of 5.5 mm was 333  μm, which agrees with the measurement. After assembly, the output energy was 41  μJ at a current of 35 A. Although the energy was lower than that of the first subprobe, the decreased beam diameter resulted in an increased fluence by a factor of >4.

### 3.7 Phantom Imaging Using Subprobes

The $2\times 2\;{\mathrm{mm}}^{2}$ single-element FP sensor was used to collect photoacoustic images from a phantom consisting of three pencil leads (300  μm diameter) separated by 1 mm using the 105  μm MMF subprobe. The MIP images of the 3D dataset in the XY, XZ, and YZ planes are shown in Figs. S6(a)–S6(c) in the Supplementary Material. The image field-of-view (FOV) was $4\times 4\;{\mathrm{mm}}^{2}$ with a scan step of 0.05 mm, and the laser repetition rate was 10 Hz. The raw A-line data were processed with a Hilbert transform to extract the envelope of the depth data, and all of the A-lines were arranged pixel by pixel to form a 3D matrix for image visualization. The total image acquisition time was about 30 min. The target-to-background ratios (T/B ratio) from the XY, XZ, and YZ images were 4.02, 5.83, and 6.45, respectively. The 25  μm MMF subprobe provided higher fluence and was used to image the second phantom consisting of 3 (42 AWG) wires (63  μm diameter) separated by 1 mm. The MIP images of the 3D dataset in the XY, XZ, and XZ planes are shown in Figs. S6(d)–S6(f) in the Supplementary Material. The T/B ratios from the XY, XZ, and XZ images were 1.65, 2.74, and 1.92, respectively.

### 3.8 In Vivo Imaging of Nude Mouse Ear

**Fig. 7:**
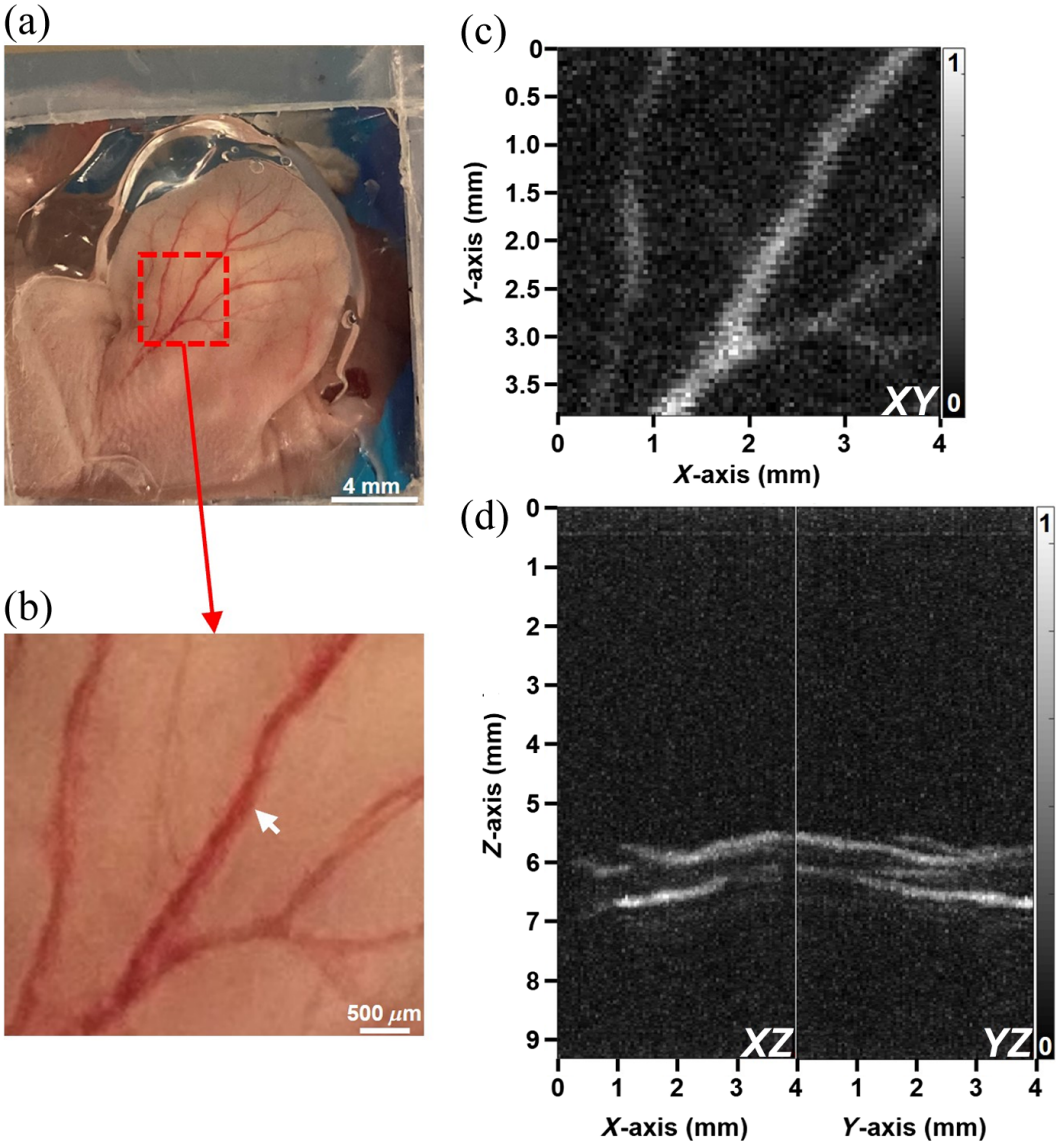
*In vivo* photoacoustic imaging. (a) Photo collected from the ear of a live mouse is shown. (b) Expanded view (red dashed square) shows outline of blood vessels (arrow). MIP images are shown in the (c) XY, and (d) XZ and YZ planes.

*In vivo* imaging of blood vessels from the ear of a live mouse was performed using excitation delivered by the 25  μm MMF subprobe and $2\times 2\;{\mathrm{mm}}^{2}$ single-element FP sensor. A photo of the mouse ear along with an expanded view is shown in Figs. 7(a) and 7(b). The MIP images of the 3D dataset in the XY, XZ, and YZ planes are shown in Figs. 7(c) and 7(d). The structure of distinct blood vessels can be clearly distinguished. The FOV was $4\times 4\;{\mathrm{mm}}^{2}$ with a pixel size of 0.05 mm, and images were collected in about 30 min. The T/B ratios from the XY, XZ, and YZ image were 2.74, 3.23, and 3.22, respectively.

## 4 Discussion

PAE is an emerging imaging modality that requires use of a miniature sensor that is highly sensitive to acoustic waves. Previous FP sensor designs have been limited by size, scalability, and array configurability for effective use in PAE. Here we addressed the physical challenges faced in scaling down the dimensions of an FP sensor and demonstrated a $2\times 2\;{\mathrm{mm}}^{2}$ prototype. A transfer matrix model was developed to describe the frequency response of a multilayer sensor, and an analytical model was derived to describe the effects of a finite thickness substrate. FEA was performed to characterize the temporal response of a finite dimensional sensor. Back filtering was performed to remove echoes in the time domain signal caused by the finite thickness of the substrate. Side waves were eliminated by surface averaging using an interrogation beam with an adequate diameter. Miniature FP sensors were fabricated using gold films as reflective mirrors deposited on either side of a parylene C film on a glass wafer. A single-wavelength laser was used to interrogate the sensor using illumination delivered by fiber subprobes. The fabricated FP sensor produced an NEP=0.76  kPa, bandwidth of 16.6 MHz, a spectral FWHM = 0.2886 nm, and quality factor Q=2694. Photoacoustic images were collected from phantoms and blood vessels in a live mouse.

The dynamic properties of an FP sensor with finite dimensions using a thin substrate to transmit pressure waves were rigorously evaluated using transfer matrix and analytical models. The results revealed a different frequency response pattern from the well-developed model for infinitely thick sensors.^12^^,^^19^ The multiple reflections incurred by propagating waves in a substrate with finite thickness form standing waves at certain frequencies, leading to zeros in the frequency response (Figs. 1 and 2). In photoacoustic imaging, the ultrasound signal is acoustic that generate echoes inside the substrate, which introduce unwanted artifacts in the time domain signal. The models in this study further revealed that the frequency response of a thin substrate-based sensor differed from that of an infinitely thick sensor only by an explicit frequency filter determined by materials properties. This finding provides the foundation for removing echoes by back filtering the time domain signal.

Simulations were also performed using FEA to investigate the boundary effects from finite sensor dimensions. Side waves that propagate toward the sensor center introduced unwanted changes in parylene C thickness. These waves originated from vibrational modes that were perpendicular to the incident acoustic waves. As further shown in the Supplementary Material, the magnitudes of such waves can be affected by various factors, such as the thickness of the substrate, the lateral size of the sensor, and the thickness of the parylene C film. Fortunately, these side waves were effectively eliminated by averaging using an interrogation beam with increased diameter. Shear waves were previously modeled in an infinitely wide planar sensor with semi-infinitely thick substrate to study the frequency-dependent directivity.^20^ The effects of coating material properties^21^ and optical conditions^22^^–^^24^ were also investigated. The findings in this study move the field forward by showing that artifacts introduced from scaling down the dimensions of an FP sensor, such as echoes and side waves, can be mitigated and that the use of this design for use in a PAE is feasible.

FP sensors with dimensions of $10\times 10\;{\mathrm{mm}}^{2}$ were fabricated first. Surface sensitivity measurements showed fringes that reflect nonuniform sensitivity using a fixed wavelength interrogation laser. However, smaller regions ($2\times 2\;{\mathrm{mm}}^{2}$) with relative uniform sensitivity could be identified. Thus a single-wavelength laser can be used for interrogation to simplify the design without sacrificing sensitivity. For endoscopic applications, a 1D array is sufficient to form images in the vertical plane without a scanning mechanism. The miniature sensors showed a NEP of 0.76 kPa. This result was not as good as larger FP sensors previously reported.^13^^,^^14^ However, the ITF and measured quality factor were comparable to the reported planar fused silica-based FP sensor.^14^ The NEP can be further improved by reducing noise in the interrogation laser and PD and with better shielding. Transparent dielectric mirrors can both improve the NEP and allow the excitation laser to pass through and provide a more compact design. The measured bandwidth was only ∼82% of the target. Since there was a lack of high-frequency components of the ultrasound wave generated by the 10 MHz transducer, the sensor response at higher frequencies may be limited. This result can be improved using a transducer with a larger bandwidth. Sensor bandwidth can be increased using a polymer film with a higher Young’s modulus and with a matched acoustic impedance.

Limitations in the design and fabrication of the miniature FP sensors will be addressed in future work. The transfer matrix and analytical models used pressure waves represented by planes travelling in the X axis only. Greater directivity can be further investigated using waves incident at an angle. In the analytical model, the acoustic impedance of the air was neglected and can be analyzed more quantitatively. In both models, the material properties were modeled as homogeneous and isotropic, but they actually are more complex in reality. In the FEA simulations, a 2D model and fixed boundary conditions were used, and more realistic models and boundary conditions can be included. Back filtering was shown to reduce the echoes in the substrate, and more advanced algorithms can be developed to better eliminate these artifacts. Gold thin films were used as reflective mirrors to achieve high reflectivity and sensitivity as proof of concept. However, these coatings are not transparent to light. Thus the excitation laser had to be placed at an angle to the sensor, thus increasing the distance to the image plane. The increased beam travel length and angled illumination reduced overall sensitivity. The bandwidth of the sensor can be further increased by decreasing the thickness of the parylene C film by sacrificing sensitivity. Thus a balance between the bandwidth and the detection sensitivity should be carefully considered in the future work.

Fiber-based FP sensors have been demonstrated and offer advantages in terms of miniature dimensions and high sensitivity.^14^^–^^18^^,^^26^ However, a tunable interrogation laser is required because the thickness of the polymer spacer cannot be accurately controlled during deposition and increases image acquisition time. The reduced lateral dimensions can introduce artifacts in the time domain signals. By comparison, our wafer-based sensor uses a single wavelength to simplify design and reduce cost. Although fringes can appear from the nonuniform thickness of the parylene film, usable areas can be found with relatively uniform sensitivity. The wafers are thin and can be diced into sensors with small dimensions for use in photoacoustic endoscopy. Many sensors that use the same wavelength can be mass manufactured.

In summary, we have demonstrated analytical and numerical models to describe the performance of a miniature FP sensor and feasibility for miniaturization. Fabrication results have verified the simulation results and showed feasibility to produce miniature wafer-based sensors. Phantom imaging and *in vivo* mouse imaging have further demonstrated the potential for using such sensors in PAE. The models have served as a powerful tool to accurately quantify sensor performance and provided theoretical guidance for design and signal processing. These findings have contributed to filling the knowledge gap in modeling miniature sensors with finite dimensions and achieving a deeper understanding of mechanisms for miniature FP sensors. The feasibility of forming a miniature transparent sensor array with single-wavelength interrogation provides an excellent choice for use in PAE applications.

## Data Availability

The data can be available from the corresponding author upon request.

## References

[1] Bell A. G., “Upon the production and reproduction of sound by light,” J. Soc. Telegr. Eng. 9(34), 404–426 (1880). 10.1049/jste-1.1880.0046

[r2] Attia A. B. E. et al., “A review of clinical photoacoustic imaging: current and future trends,” Photoacoustics 16, 100144 (2019). 10.1016/j.pacs.2019.100144 31871888 PMC6911900

[3] Wang L. V. Yao J., “A practical guide to photoacoustic tomography in the life sciences,” Nat. Methods 13(8), 627–638 (2016).1548-7091 10.1038/nmeth.3925 27467726 PMC4980387

[4] Kim M. et al., “Intra-instrument channel workable, optical-resolution photoacoustic and ultrasonic mini-probe system for gastrointestinal endoscopy,” Photoacoustics 26, 100346 (2022). 10.1016/j.pacs.2022.100346 35313458 PMC8933520

[r5] Li Y. et al., “High-speed integrated endoscopic photoacoustic and ultrasound imaging system,” IEEE J. Sel. Top. Quantum Electron. 25(1), 7102005 (2019). IJSQEN 1077-260X 10.1109/JSTQE.2018.2869614 31447542 PMC6707714

[r6] Zhu Y. et al., “Prototype endoscopic photoacoustic-ultrasound balloon catheter for characterizing intestinal obstruction,” Biomed. Opt. Express 13(6), 3355–3365 (2022). BOEICL 2156-7085 10.1364/BOE.456672 35781972 PMC9208587

[r7] Leng J. et al., “Multi-spectral intravascular photoacoustic/ultrasound/optical coherence tomography tri-modality system with a fully-integrated 0.9-mm full field-of-view catheter for plaque vulnerability imaging,” Biomed. Opt. Express 12(4), 1934–1946 (2021). BOEICL 2156-7085 10.1364/BOE.420724 33996208 PMC8086469

[r8] Xiong K. et al., “Autofocusing optical-resolution photoacoustic endoscopy,” Opt. Lett. 43(8), 1846–1849 (2018). OPLEDP 0146-9592 10.1364/OL.43.001846 29652380

[r9] Liu N. Yang S. Xing D., “Photoacoustic and hyperspectral dual-modality endoscope,” Opt. Lett. 43(1), 138–141 (2018). OPLEDP 0146-9592 10.1364/OL.43.000138 29328216

[10] Lin R. et al., “Miniature intravascular photoacoustic endoscopy with coaxial excitation and detection,” J. Biophotonics 16, e202200269 (2023). 10.1002/jbio.202200269 36510391

[11] Li Y. et al., “Advances in endoscopic photoacoustic imaging,” Photonics 8(7), 281 (2021). 10.3390/photonics8070281 35252433 PMC8896876

[r12] Beard P. C. Perennes F. Mills T. N., “Transduction mechanisms of the Fabry-Perot polymer film sensing concept for wideband ultrasound detection,” IEEE Trans. Ultrasonics, Ferroelectr. Freq. Control 46(6), 1575–1582 (1999). 10.1109/58.808883 18244356

[13] Zhang E. Laufer J. Beard P., “Backward-mode multiwavelength photoacoustic scanner using a planar Fabry-Perot polymer film ultrasound sensor for high-resolution three-dimensional imaging of biological tissues,” Appl. Opt. 47(4), 561–577 (2008). APOPAI 0003-6935 10.1364/AO.47.000561 18239717

[14] Guggenheim J. A. et al., “Ultrasensitive plano-concave optical microresonators for ultrasound sensing,” Nat. Photonics 11(11), 714–719 (2017). NPAHBY 1749-4885 10.1038/s41566-017-0027-x

[r15] Mezil S. et al., “Single-shot hybrid photoacoustic-fluorescent microendoscopy through a multimode fiber with wavefront shaping,” Biomed. Opt. Express 11(10), 5717–5727 (2020). BOEICL 2156-7085 10.1364/BOE.400686 33149981 PMC7587274

[r16] Mathews S. J. et al., “All-optical dual photoacoustic and optical coherence tomography intravascular probe,” Photoacoustics 11, 65–70 (2018). 10.1016/j.pacs.2018.07.002 30112279 PMC6092552

[r17] Ansari R. et al., “All-optical forward-viewing photoacoustic probe for high-resolution 3D endoscopy,” Light Sci. Appl. 7(1), 75 (2018). 10.1038/s41377-018-0070-5 30323927 PMC6177463

[18] Ansari R. et al., “Miniature all-optical flexible forward-viewing photoacoustic endoscopy probe for surgical guidance,” Opt. Lett. 45(22), 6238–6241 (2020). OPLEDP 0146-9592 10.1364/OL.400295 33186959 PMC8219374

[19] Beard P. C. Mills T. N., “Extrinsic optical-fiber ultrasound sensor using a thin polymer film as a low-finesse Fabry–Perot interferometer,” Appl. Opt. 35(4), 663–675 (1996). APOPAI 0003-6935 10.1364/AO.35.000663 21069054

[20] Cox B. T. Beard P. C., “The frequency-dependent directivity of a planar Fabry-Perot polymer film ultrasound sensor,” IEEE Trans. Ultrasonics, Ferroelectr. Freq. Control 54(2), 394–404 (2007). 10.1109/TUFFC.2007.253 17328336

[21] Buchmann J. et al., “Characterization and modeling of Fabry–Perot ultrasound sensors with hard dielectric mirrors for photoacoustic imaging,” Appl. Opt. 56, 5039 (2017). APOPAI 0003-6935 10.1364/AO.56.005039 29047652

[22] Marques D. M. et al., “Modelling Fabry-Pérot etalons illuminated by focussed beams,” Opt. Express 29(5), 7691–7706 (2020). OPEXFF 1094-4087 10.1364/OE.382526 32225991

[r23] Marques D. M. Guggenheim J. A. Munro P. R. T., “Angular Airy function: a model of Fabry-Perot etalons illuminated by arbitrary beams,” Opt. Express 29(15), 24144–24150 (2021). OPEXFF 1094-4087 10.1364/OE.431362 34614664

[24] Marques D. M. Guggenheim J. A. Munro P. R. T., “Analysing the impact of non-parallelism in Fabry-Perot etalons through optical modelling,” Opt. Express 29(14), 21603–21614 (2021). OPEXFF 1094-4087 10.1364/OE.425487 34265944

[25] Stancik A. L. Brauns E. B., “A simple asymmetric lineshape for fitting infrared absorption spectra,” Vib. Spectrosc. 47(1), 66–69 (2008). VISPEK 0924-2031 10.1016/j.vibspec.2008.02.009

[26] Guo Z. Li G. Chen S. L.. “Miniature probe for all-optical double gradient-index lenses photoacoustic microscopy,” J. Biophotonics 11(12), e201800147 (2018). 10.1002/jbio.201800147 30003707

