# Supplementary material for: Scaling down the dimensions of a Fabry–Perot polymer film acoustic sensor for photoacoustic endoscopy
Source: J Biomed Opt. 2024 Jan 2;29(Suppl 1):S11514. doi: 10.1117/1.JBO.29.S1.S11514 (PMC10760494; doi:10.1117/1.JBO.29.S1.S11514)
Supplement: Supplementary file 1 [file JBO_029_S11514_SD001.pdf]

Supplementary Material

Detailed Derivation of Transfer Matrix Model

A mathematical model was developed to characterize the frequency response of the miniature FP sensor to an incident pressure wave, Fig. S1 (a). The pressure waves on either side of each interface must meet the boundary conditions:

$$\begin{bmatrix} P(x=0) \\ v(x=0) \end{bmatrix} = \begin{bmatrix} e^{-ik_2 0} & e^{ik_2 0} \\ \frac{1}{z_2} e^{-ik_2 0} & -\frac{1}{z_2} e^{ik_2 0} \end{bmatrix} \begin{bmatrix} P_1 \\ P_2 \end{bmatrix} = \begin{bmatrix} e^{-ik_1 0} & e^{ik_1 0} \\ \frac{1}{z_1} e^{-ik_1 0} & -\frac{1}{z_1} e^{ik_1 0} \end{bmatrix} \begin{bmatrix} P_0 \\ P_R \end{bmatrix}, \quad (10)$$

$$\begin{bmatrix} P(x=l) \\ v(x=l) \end{bmatrix} = \begin{bmatrix} e^{-ik_3 l} & e^{ik_3 l} \\ \frac{1}{z_3} e^{-ik_3 l} & -\frac{1}{z_3} e^{ik_3 l} \end{bmatrix} \begin{bmatrix} P_3 \\ P_4 \end{bmatrix} = \begin{bmatrix} e^{-ik_2 l} & e^{ik_2 l} \\ \frac{1}{z_2} e^{-ik_2 l} & -\frac{1}{z_2} e^{ik_2 l} \end{bmatrix} \begin{bmatrix} P_1 \\ P_2 \end{bmatrix}, \quad (11)$$

$$\begin{bmatrix} P(x=l+H) \\ v(x=l+H) \end{bmatrix} = \begin{bmatrix} e^{-ik_4(l+H)} & e^{ik_4(l+H)} \\ \frac{1}{z_4} e^{-ik_4(l+H)} & -\frac{1}{z_4} e^{ik_4(l+H)} \end{bmatrix} \quad (12)$$

$$\begin{bmatrix} P_T \\ 0 \end{bmatrix} = \begin{bmatrix} e^{-ik_3(l+H)} & e^{ik_3(l+H)} \\ \frac{1}{z_3} e^{-ik_3(l+H)} & -\frac{1}{z_3} e^{ik_3(l+H)} \end{bmatrix} \begin{bmatrix} P_3 \\ P_4 \end{bmatrix}, \quad (13)$$

where P(x) and v(x) are the pressure and velocity, respectively, at position x. The wave numbers, k_1, k_2, k_3 and k_4, characterize the pressure waves in tissue, parylene C, glass and air, respectively, and are defined as: $k_1 = \frac{\omega}{c_1}, k_2 = \frac{\omega}{c_2}, k_1 = \frac{\omega}{c_3}, k_1 = \frac{\omega}{c_4}$, where ω is the angular frequency of the wave, c_1, c_2, c_3 and c_4 are the speeds of sound in tissue, parylene C, glass and air, respectively.

From these equations, we have:

$$\begin{bmatrix} P_1 \\ P_2 \end{bmatrix} = \begin{bmatrix} e^{-ik_2 0} & e^{ik_2 0} \\ \frac{1}{z_2} e^{-ik_2 0} & -\frac{1}{z_2} e^{ik_2 0} \end{bmatrix}^{-1} \begin{bmatrix} e^{-ik_1 0} & e^{ik_1 0} \\ \frac{1}{z_1} e^{-ik_1 0} & -\frac{1}{z_1} e^{ik_1 0} \end{bmatrix} \quad (14)$$

$$\begin{bmatrix} P_0 \\ P_R \end{bmatrix} = \begin{bmatrix} 1 & 1 \\ \frac{1}{z_2} & -\frac{1}{z_2} \end{bmatrix}^{-1} \begin{bmatrix} 1 & 1 \\ \frac{1}{z_1} & -\frac{1}{z_1} \end{bmatrix} \begin{bmatrix} P_0 \\ P_R \end{bmatrix}, \quad (15)$$

$$\begin{bmatrix} P_3 \\ P_4 \end{bmatrix} = \begin{bmatrix} e^{-ik_3 l} & e^{ik_3 l} \\ \frac{1}{z_3} e^{-ik_3 l} & -\frac{1}{z_3} e^{ik_3 l} \end{bmatrix}^{-1} \begin{bmatrix} e^{-ik_2 l} & e^{ik_2 l} \\ \frac{1}{z_2} e^{-ik_2 l} & -\frac{1}{z_2} e^{ik_2 l} \end{bmatrix} \begin{bmatrix} P_1 \\ P_2 \end{bmatrix}, \tag{16}$$

$$\begin{bmatrix} P_T \\ 0 \end{bmatrix} = \begin{bmatrix} e^{-ik_4(l+H)} & e^{ik_4(l+H)} \\ \frac{1}{z_4} e^{-ik_4(l+H)} & -\frac{1}{z_4} e^{ik_4(l+H)} \end{bmatrix}^{-1} \begin{bmatrix} e^{-ik_3(l+H)} & e^{ik_3(l+H)} \\ \frac{1}{z_3} e^{-ik_3(l+H)} & -\frac{1}{z_3} e^{ik_3(l+H)} \end{bmatrix} \begin{bmatrix} P_3 \\ P_4 \end{bmatrix}, \tag{17}$$

Thus,

$$\begin{bmatrix} P_T \\ 0 \end{bmatrix} = \begin{bmatrix} e^{-ik_4(l+H)} & e^{ik_4(l+H)} \\ \frac{1}{z_4} e^{-ik_4(l+H)} & -\frac{1}{z_4} e^{ik_4(l+H)} \end{bmatrix}^{-1} \times \begin{bmatrix} e^{-ik_3(l+H)} & e^{ik_3(l+H)} \\ \frac{1}{z_3} e^{-ik_3(l+H)} & -\frac{1}{z_3} e^{ik_3(l+H)} \end{bmatrix} \times$$

$$\begin{bmatrix} e^{-ik_3 l} & e^{ik_3 l} \\ \frac{1}{z_3} e^{-ik_3 l} & -\frac{1}{z_3} e^{ik_3 l} \end{bmatrix}^{-1} \times \begin{bmatrix} e^{-ik_2 l} & e^{ik_2 l} \\ \frac{1}{z_2} e^{-ik_2 l} & -\frac{1}{z_2} e^{ik_2 l} \end{bmatrix} \times \begin{bmatrix} 1 & 1 \\ \frac{1}{z_2} & -\frac{1}{z_2} \end{bmatrix}^{-1} \times \begin{bmatrix} 1 & 1 \\ \frac{1}{z_1} & -\frac{1}{z_1} \end{bmatrix} \times \begin{bmatrix} P_0 \\ P_R \end{bmatrix} =$$

$$\begin{bmatrix} M_{11} & M_{12} \\ M_{21} & M_{22} \end{bmatrix} \begin{bmatrix} P_0 \\ P_R \end{bmatrix} = M \begin{bmatrix} P_0 \\ P_R \end{bmatrix}, \tag{18}$$

where $M = \begin{bmatrix} M_{11} & M_{12} \\ M_{21} & M_{22} \end{bmatrix}$ is defined as the system transfer matrix.

Detailed Derivation of Analytical Model

An analytical model was developed to describe the pressure waves undergoing multiple reflections inside the sensor substrate, Fig. S1 (b). The pressure wave P_0 is incident on the parylene C film and induces a change in thickness that is transmitted as P_1 into the glass substrate. P_1 is reflected at the glass-air interface, and travels back toward the parylene C film. P_1 is reflected again at the glass-paryelene C interface and induces a secondary thickness change. This pressure wave is reflected repeatedly inside the glass substrate resulting in an echo. T represents the acoustic transmission coefficient through the parylene C film. R_1 represents the reflection coefficient between air and an infinitely thick glass substrate. R_2 represents the reflection coefficient between an infinite glass substrate and a parylene C film with thickness *l*. The transmission coefficient T

can be derived explicitly using the transfer matrix method, presented above, when the glass substrate is infinitely thick, and is given by:

$$T = \frac{2z_3}{z_1} e^{ik_3 l} \frac{1}{\left(1+\frac{z_3}{z_1}\right)\cos(k_2 l)+i\left(\frac{z_2}{z_1}+\frac{z_3}{z_2}\right)\sin(k_2 l)}, \tag{19}$$

Similarly, the expressions for R_1 and R_2 are given by:

$$R_1 \approx -e^{-2ik_3(H+l)} \tag{20}$$

$$R_2 = -e^{2ik_3 l} \frac{\left(1-\frac{z_1}{z_3}\right)\cos(k_2 l)+i\left(-\frac{z_2}{z_3}+\frac{z_1}{z_2}\right)\sin(k_2 l)}{\left(1+\frac{z_1}{z_3}\right)\cos(k_2 l)+i\left(\frac{z_2}{z_3}+\frac{z_1}{z_2}\right)\sin(k_2 l)}, \tag{21}$$

Note that the expression for R_1 can be approximated by assuming that the acoustic impedance of air is much smaller than that of glass. Since the reflected pressure wave is incident at the glass-parylene C interface from the glass side, the induced thickness change for the parylene C film will be different from that from the tissue side. The thickness change for parylene C induced by a unit pressure wave from the tissue side is:

$$\Delta l_0 = -\frac{4\sin\left(\frac{1}{2}k_2 l\right)}{\omega z_2} \frac{\frac{z_3}{z_1}\cos\left(\frac{1}{2}k_2 l\right)+i\frac{z_2}{z_1}\sin\left(\frac{1}{2}k_2 l\right)}{\left(1+\frac{z_3}{z_1}\right)\cos(k_2 l)+i\left(\frac{z_2}{z_1}+\frac{z_3}{z_2}\right)\sin(k_2 l)}, \tag{22}$$

Note this expression is actually the frequency response F(ω) of the FP sensor with an infinitely thick substrate. Similarly, the thickness change induced by a unit pressure wave from the glass side is:

$$\Delta l_1 = -\frac{4\sin\left(\frac{1}{2}k_2 l\right)}{\omega z_2} e^{ik_3 l} \frac{\frac{z_1}{z_3}\cos\left(\frac{1}{2}k_2 l\right)+i\frac{z_2}{z_3}\sin\left(\frac{1}{2}k_2 l\right)}{\left(1+\frac{z_1}{z_3}\right)\cos(k_2 l)+i\left(\frac{z_2}{z_3}+\frac{z_1}{z_2}\right)\sin(k_2 l)}, \tag{23}$$

Define η to be the ratio between the thickness changes caused by incident waves from the glass and tissue sides. Thus,

$$\eta = \frac{\Delta l_1}{\Delta l_0} = e^{ik_3 l} \frac{\frac{z_1}{z_3}\cos\left(\frac{1}{2}k_2 l\right)+i\frac{z_2}{z_3}\sin\left(\frac{1}{2}k_2 l\right)}{\left(1+\frac{z_1}{z_3}\right)\cos(k_2 l)+i\left(\frac{z_2}{z_3}+\frac{z_1}{z_2}\right)\sin(k_2 l)} \frac{\left(1+\frac{z_3}{z_1}\right)\cos(k_2 l)+i\left(\frac{z_2}{z_1}+\frac{z_3}{z_2}\right)\sin(k_2 l)}{\frac{z_3}{z_1}\cos\left(\frac{1}{2}k_2 l\right)+i\frac{z_2}{z_1}\sin\left(\frac{1}{2}k_2 l\right)}, \tag{24}$$

The total pressure P_G inside of the glass is the sum of all the reflected pressure waves, and can be calculated as:

$$P_G = P^{(1)} + P^{(2)} + P^{(3)} \ldots = P^{(1)} + P^{(1)}R_1R_2 + P^{(1)}(R_1R_2)^2 + \cdots = P^{(1)}\sum_{n=0}^{\infty}(R_1R_2)^n$$

$$= P^{(1)}\frac{1}{1-R_1R_2} = P_0TR_1\frac{1}{1-R_1R_2}, \qquad (25)$$

Now the total thickness change of a sensor with a glass substrate, Δl, can be written as the sum of the thickness changes Δl_0 induced by P_0 and Δl_G induced by P_G:

$$\Delta l = \Delta l_0 + \Delta l_G \qquad (26)$$

Since Δl_0 is proportional to P_0 and Δl_G is proportional to P_G, using η defined as:

$$\Delta l = \Delta l_0 + \Delta l_0 TR_1\eta\frac{1}{1-R_1R_2} = \Delta l_0\left(1 + \frac{TR_1\eta}{1-R_1R_2}\right) \qquad (27)$$

Thus Δl can be written as $\Delta l(\omega) = F_H(\omega)\Delta l_0(\omega)$, where $F_H(\omega) = 1 + \frac{TR_1\eta}{1-R_1R_2}$ is defined as a frequency domain filter that converts the original sensor frequency response $\Delta l_0(\omega)$ to that of the new finite thickness sensor $\Delta l(\omega)$. After including expressions for the coefficients and simplifying the result, the explicit expression for $F_H(\omega)$ is:

$$F_H(\omega) = \frac{1 - e^{-2ik_3H}\frac{z_3\cos\left(\frac{1}{2}k_2l\right) - iz_2\sin\left(\frac{1}{2}k_2l\right)}{z_3\cos\left(\frac{1}{2}k_2l\right) + iz_2\sin\left(\frac{1}{2}k_2l\right)}}{1 - e^{-2ik_3H}\frac{\left(1-\frac{z_1}{z_3}\right)\cos(k_2l) + i\left(-\frac{z_2}{z_3}+\frac{z_1}{z_2}\right)\sin(k_2l)}{\left(1+\frac{z_1}{z_3}\right)\cos(k_2l) + i\left(\frac{z_2}{z_3}+\frac{z_1}{z_2}\right)\sin(k_2l)}}, \qquad (28)$$

Hence, the frequency response of a finite thickness sensor is simply that of an infinitely thick sensor multiplied by $F_H(\omega)$.

Finite element analysis simulations

Additional parameters were analyzed to characterize the effect of side waves in the miniature FP sensor with finite dimensions. The first parameter was the thickness of the glass substrate. The change in thickness is shown at the center of parylene C film with 2 mm length and 32 µm

thickness coupled to 100, 200, and 500 μm thick glass substrates, Fig. S4 (a)-(c). The magnitude of the side waves was comparable to that of the main signal and created an undesirable artifact. Standard deviations after subtracting out the main signal from an infinitely wide sensor were 0.127, 0.157 and 0.164, respectively. This result shows that the thickness of the glass did not affect the side wave significantly. The side wave depends more on the parameters of the parylene C film, while the substrate thickness affects the multi-reflection echo signals.

The second parameter studied was the lateral width of the FP sensor. The change in thickness is shown at the center of parylene C film with 32 μm thickness coupled to a 500 μm thick glass substrate for 1, 2, and 3 mm widths, Fig. S4 (d)-(f). Side waves appeared later when the lateral size of the sensor was larger as time is required for the waves to travel to the sensor center. Also, the magnitude of the side waves was smaller for wider sensors because of less stiffness. Standard deviations after subtracting out the main signal from an infinitely wide sensor were 0.198, 0.164 and 0.090, respectively. Thus, a sensor with a larger lateral dimensions will reduce the magnitude of the side waves.

The third parameter studied was the thickness of the parylene C film. The change in thickness is shown for a 2 mm wide sensor thickness coupled to a 500 μm thick glass substrate with a parylene C film thicknesses of 12, 22, 32, and 42 μm, Fig. S4 (g)-(j). Standard deviations after subtracting out the main signal from an infinitely wide sensor were 0.050, 0.117, 0.164 and 0.171, respectively. The magnitude of the side waves decreased and became insignificant compared with that of the main signal as the thickness of the parylene C film was reduced. However, the absolute magnitude of the main signal also decreased due to the decrease in parylene C thickness. Thus, eliminating the side waves results in tradeoff of sensor sensitivity.

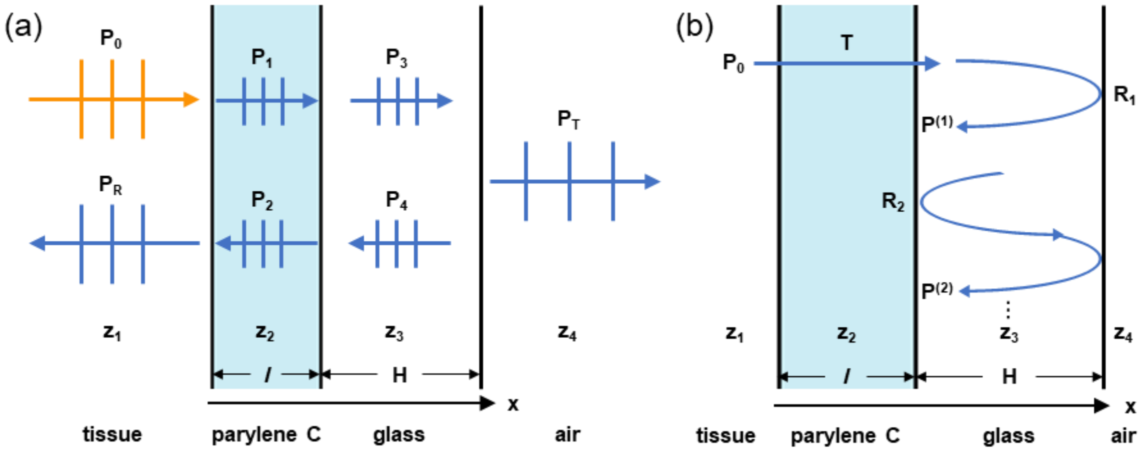

Fig. S1 Transfer matrix and analytical models. (a) P_0 and P_R represent the magnitudes of the input and reflected pressure waves, respectively, traveling in tissue. P_1 and P_2 are the magnitudes of the pressure waves traveling in the parylene C film with thickness *l*. P_3 and P_4 are the magnitudes of the pressure waves travelling in the glass substrate with thickness H. P_T is the magnitude of the transmitted pressure wave in air. z_1, z_2, z_3, and z_4 represent the acoustic impedance in tissue, parylene C, glass, and air, respectively. Each layer is assumed to have infinite lateral dimensions, and all pressure waves are assumed to be planar. (b) P_0 is the magnitude of the pressure wave incident on the parylene C film with thickness *l*. $P^{(1)}$ is the magnitude of the pressure wave transmitted into the glass substrate with thickness H. $P^{(1)}$ is reflected at the glass-air interface back toward the parylene C film, and is reflected again at the glass-paryelene C interface to become $P^{(2)}$ and so on. R_1 and R_2 are the reflection coefficients at the glass-air and glass-parylene C interfaces, respectively.

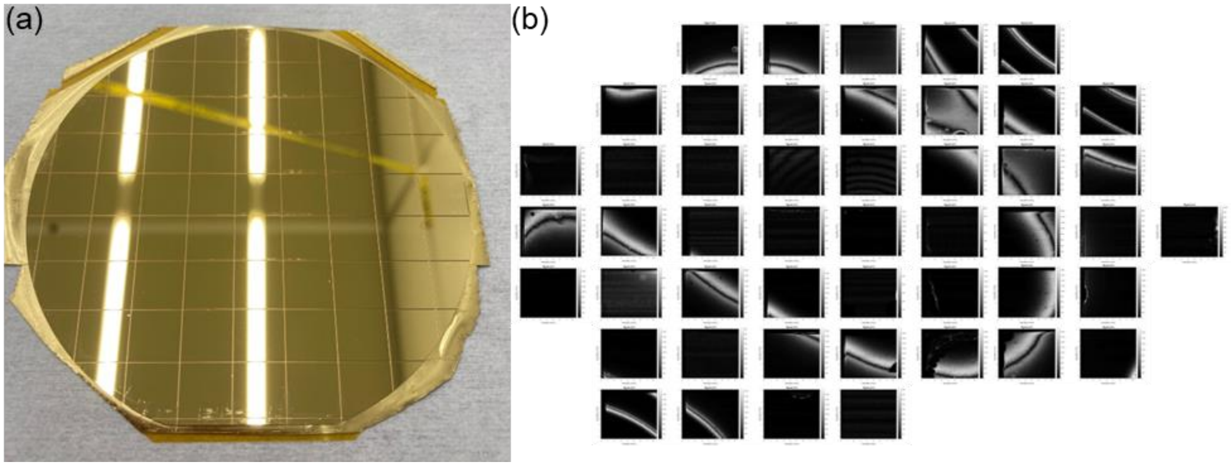

Fig. **S2** Miniature FP sensors. (a) Sensors with 10×10 mm^2 dimensions were diced from a 100 mm diameter wafer. (b) Surface sensitivities of individual sensors in (a) are measured and shown.

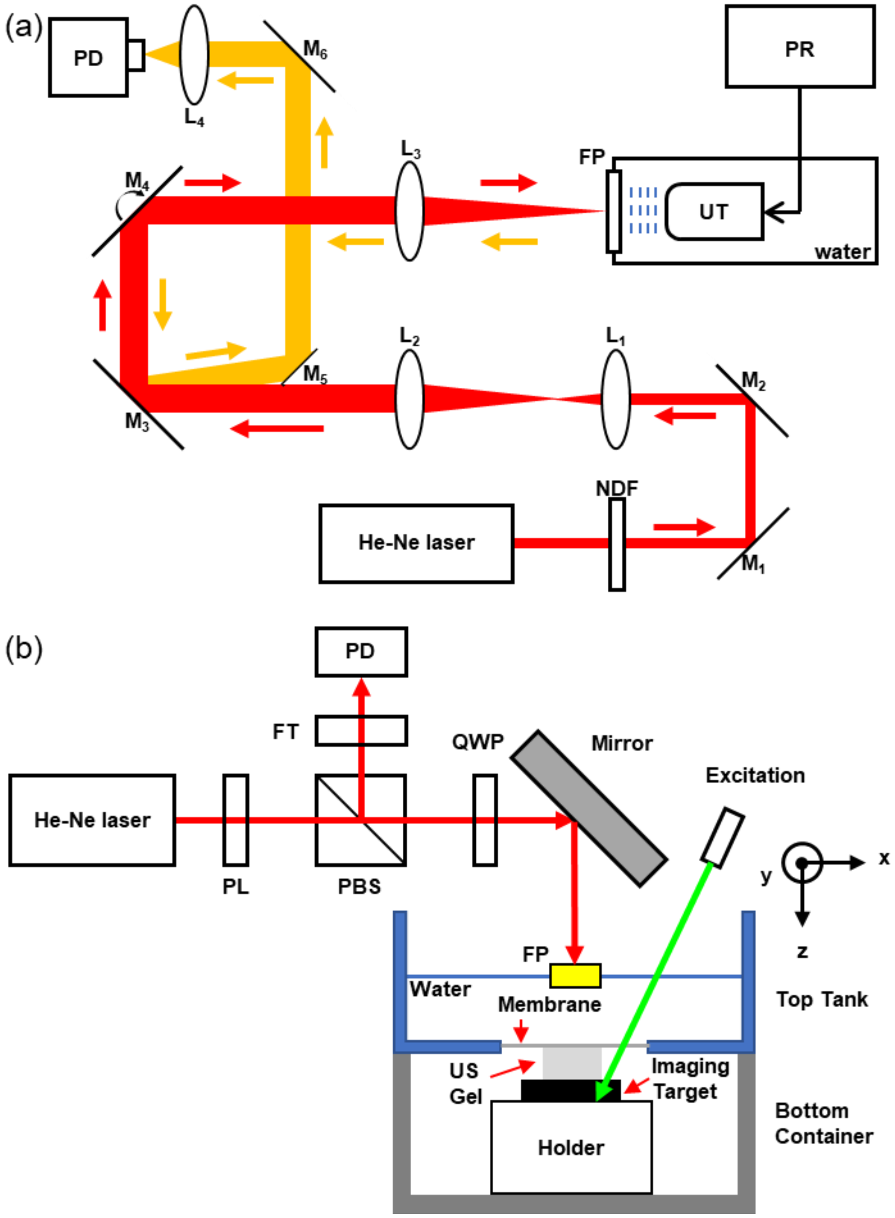

Fig. S3 Characterization of FP sensors. Schematics are shown for the tabletop photoacoustic imaging systems used to characterize the (a) 10×10 and (b) 2×2 mm^2 FP sensors, respectively. Details are provided in the text.

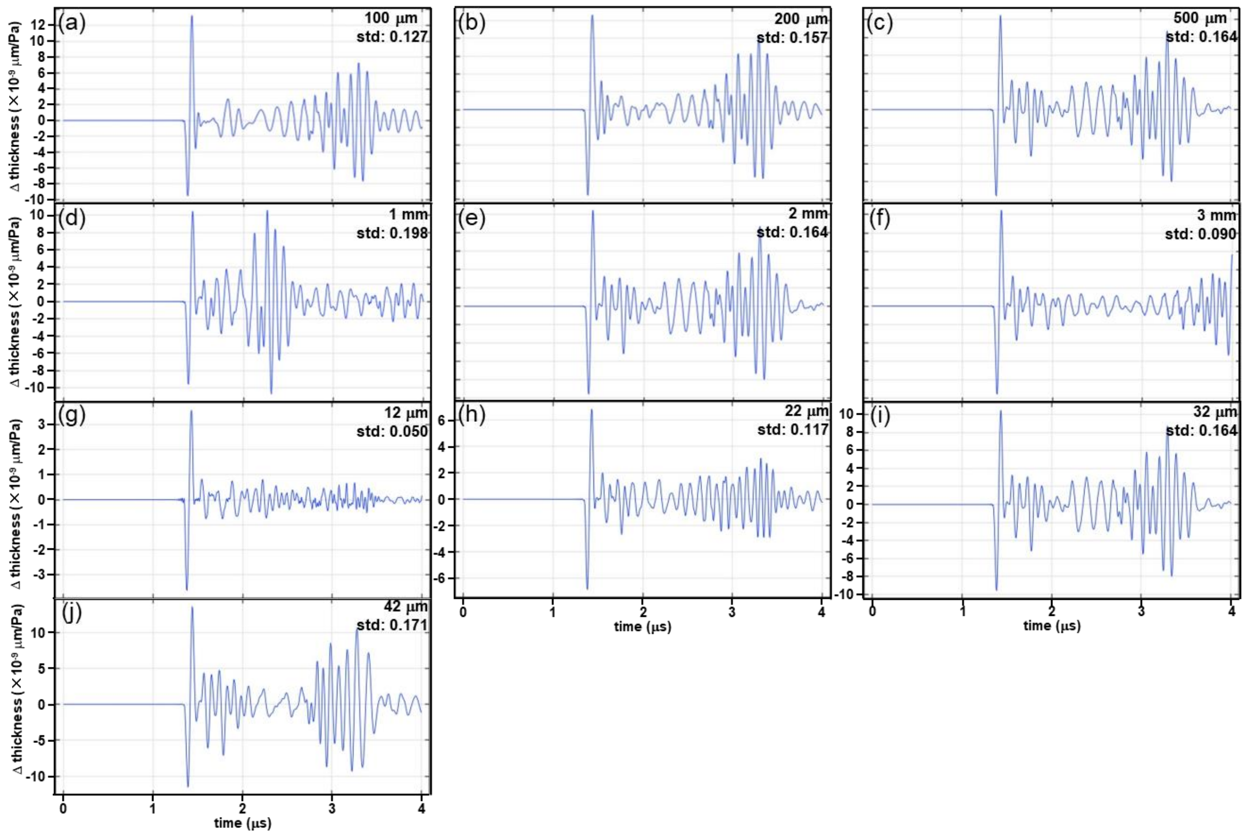

Fig. S4 Miniature FP sensor design. *Effect of substrate thickness*. The change in thickness for the parylene C film from an incident pressure wave P_0 is shown for a 2 mm wide sensor with (a) 100 µm, (b) 200 µm, (c) 500 µm thick glass substrates. The difference in signal from that of an infinitely wide sensor has a standard deviation of 0.127, 0.157 and 0.164, respectively. *Effect of lateral size of the sensor*. The change in thickness for the parylene C film from an incident pressure wave P_0 is shown for a sensor with 500 µm thick glass substrates and (d) 1 mm, (e) 2 mm, (f) 3 mm lateral size. The difference in signal from that of an infinitely wide sensor has a standard deviation of 0.198, 0.164 and 0.090, respectively. *Effect of the thickness of the parylene C film*. The change in thickness for the parylene C film from an incident pressure wave P_0 is shown for a 2 mm wide sensor with 500 µm thick glass substrates and (g) 12 µm, (h) 22 µm, (i) 32 µm and (j) 42 µm thick parylene C film. The difference in signal from that of an infinitely wide sensor has a standard deviation of 0.050, 0.117, 0.164 and 0.171, respectively.

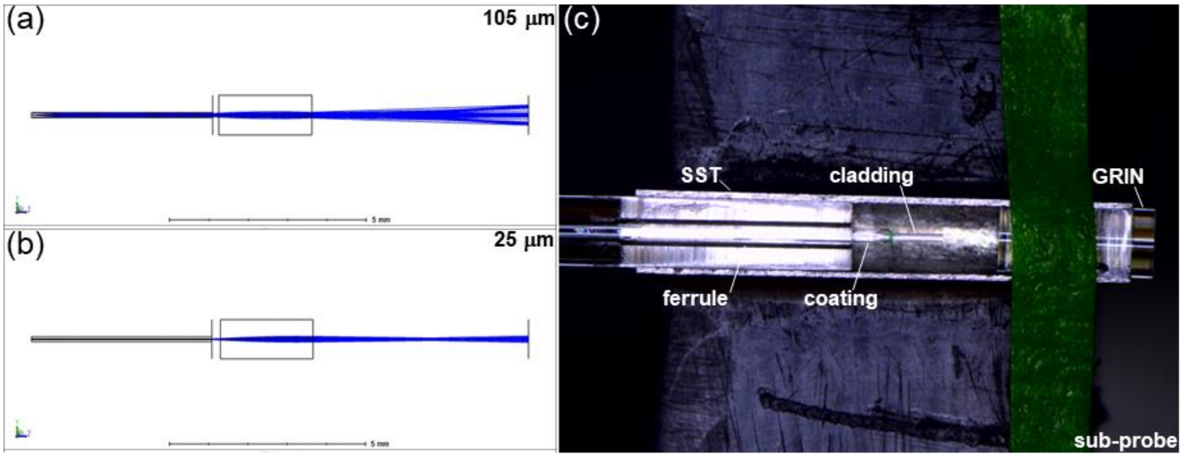

Fig. S5 Excitation sub-probes. Ray trace simulations are shown for λ = 532 nm sub-probes with (a) 105 and (b) 25 μm core MMFs used to characterize the 2×2 mm^2 FP sensors. (c) Photo shows details of optical components packaged within a stainless steel tube (SST).

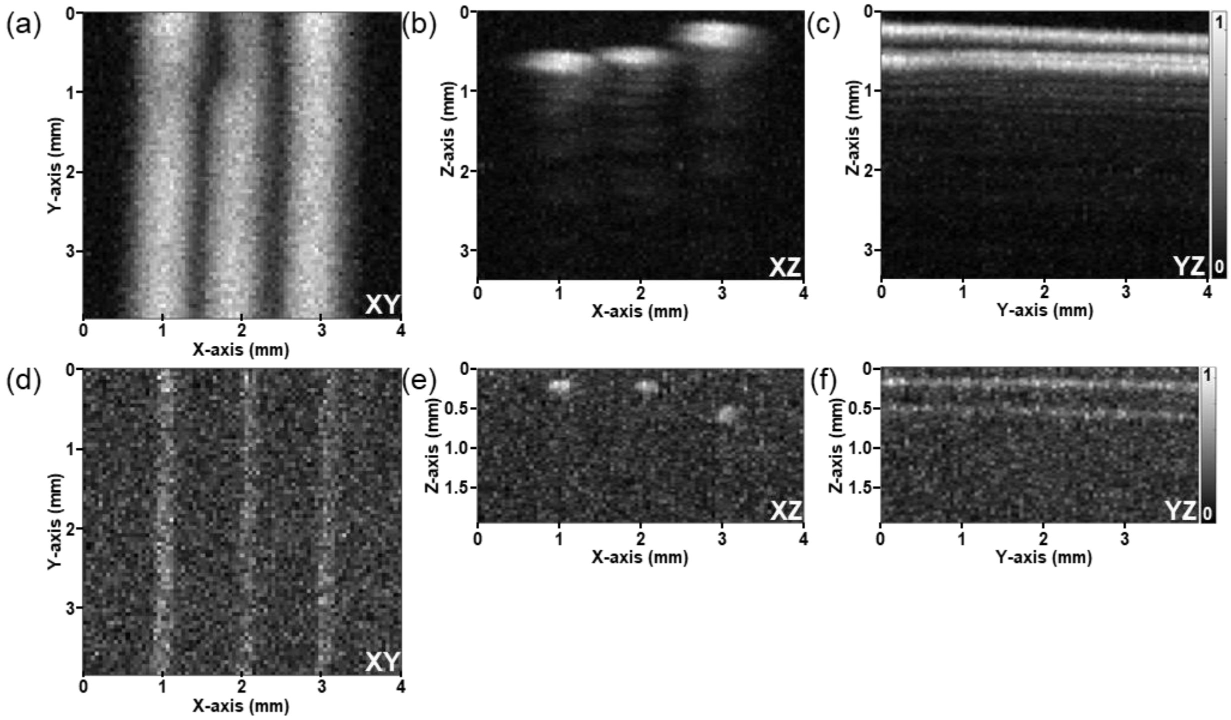

Fig. S6 Photoacoustic images of phantoms. Photoacoustic images collected with the 2×2 mm^2 FP sensors are shown from 3 pencil leads (300 μm diameter) in the (a) XY, (b) XZ, and (c) YZ planes, respectively, and of 3 (42 AWG) wires (63 μm diameter) in the (d) XY, (e) XZ, and (f) YZ planes, respectively. The phantoms were arranged 1 mm apart, and sub-probes with 105 and 25 μm core MMFs, respectively, were used.
